# Gender Differences in Adipocyte Metabolism and Liver Cancer Progression

**DOI:** 10.3389/fgene.2016.00168

**Published:** 2016-09-20

**Authors:** Otto K.-W. Cheung, Alfred S.-L. Cheng

**Affiliations:** ^1^School of Biomedical Sciences, The Chinese University of Hong Kong Hong Kong, China; ^2^State Key Laboratory of Digestive Disease, The Chinese University of Hong Kong Hong Kong, China

**Keywords:** adipocyte, adipokine, adiponectin, epigenetic, gender dimorphism, hepatocellular carcinoma, leptin, metabolism

## Abstract

Liver cancer is the third most common cancer type and the second leading cause of deaths in men. Large population studies have demonstrated remarkable gender disparities in the incidence and the cumulative risk of liver cancer. A number of emerging risk factors regarding metabolic alterations associated with obesity, diabetes and dyslipidemia have been ascribed to the progression of non-alcoholic fatty liver diseases (NAFLD) and ultimately liver cancer. The deregulation of fat metabolism derived from excessive insulin, glucose, and lipid promotes cancer-causing inflammatory signaling and oxidative stress, which eventually triggers the uncontrolled hepatocellular proliferation. This review presents the current standing on the gender differences in body fat compositions and their mechanistic linkage with the development of NAFLD-related liver cancer, with an emphasis on genetic, epigenetic and microRNA control. The potential roles of sex hormones in instructing adipocyte metabolic programs may help unravel the mechanisms underlying gender dimorphism in liver cancer and identify the metabolic targets for disease management.

## Introduction

Liver cancer is currently the third most common cancer type and the second leading cause of deaths in men (521,000, 6.4% of the total, per annum; Bray et al., 2013; Ferlay et al., 2015; GBD 2013 Mortality and Causes of Death Collaborators, 2015). Despite the use of aggressive treatments, the 5-years survival rate for patients with liver cancer only varies around 10% and will remain a global health concern, especially in Asian countries (Farazi and DePinho, 2006; Bray et al., 2013; Villanueva and Llovet, 2014). Hepatocellular carcinoma (HCC) has accounted for a majority (90%) of liver cancer cases. Risk factors leading to HCC comprise a medical history of chronic liver diseases, including liver cirrhosis, hepatitis, alcoholic and non-alcoholic fatty liver diseases (AFLD and NAFLD, respectively). NAFLD is characterized by abnormal accumulation of TAG in hepatocytes under metabolic and viral stimulation and it is reported to be one of the most prevalent causes of chronic liver diseases in Western countries, constituting an estimated prevalence of 20–40% (Tiniakos et al., 2010; Chalasani et al., 2012). NAFLD may progress into NASH, cirrhosis and ultimately HCC (Tian et al., 2013).

Emerging evidence has ascribed the development of liver cancer to obesity and type 2 diabetes (Welzel et al., 2013). The World Health Organization (WHO) categorizes a body mass index (BMI) ≥ 25 kg/m^2^ as overweight and a BMI ≥ 30 kg/m^2^ as obese. From 1980 to 2008, the worldwide prevalence of obesity has doubled, according to the statistics of WHO (Finucane et al., 2011). Obesity does not only induce cancer-causing chronic inflammation, but also cause alterations in the endocrine system (Baffy et al., 2012; Vongsuvanh et al., 2012), which might altogether contribute to the increased risks for the development of NAFLD and HCC (Vanni and Bugianesi, 2014; Chang et al., 2016). Due to the difficult differentiation in a large scale, separate global estimates of prevalence for types 1 and 2 diabetes are not available (NCD Risk Factor Collaboration [NCD-RisC], 2016). The skyrocketing increase in the number of diabetic patients estimated by WHO (fourfolds) from 1980 to 2014 has coincided with the current increase of liver cancer incidence (NCD Risk Factor Collaboration [NCD-RisC], 2016). Indeed, a recent study has as well estimated that patients with a history of diabetes and/or obesity will exhibit a 2.47-fold higher liver cancer risk (Welzel et al., 2013). The present epidemic of fat-associated metabolic syndromes is positively correlated to the increasing trend of HCC all over the world.

Sexual dimorphism is the biological inequality between males and females in disease initiation and progression. Deregulated signaling of sex hormones, i.e., androgen in males and estrogens in females, is believed to be one of the drivers of sexual dimorphism in HCC. The male-to-female ratio averages between 2:1 and 7:1 in virus and NAFLD-related HCCs (Parkin et al., 2006; Guerrero et al., 2009; El-Serag, 2012; Tian et al., 2013). Recent population-based studies have consistently demonstrated much higher risk of HCC in obese men. In a prospective study including more than 900,000 adults, it was found that men with a BMI of 35 kg/m^2^ exhibited a dramatic 4.52-fold increase in relative risk of death from liver cancer, while a modest 1.68-fold increase was observed in women (Calle et al., 2003). A cohort study of 5.24 million adults in UK has further confirmed the significant modulation of HCC incidence by gender (Bhaskaran et al., 2014). More recently, a multi-ethnic cohort study has shown that BMI was strongly associated with HCC in Japanese, white, and Latino men (Bhaskaran et al., 2014). The multi-regional epidemiological studies have all revealed the high male-to-female ratio in both HCC incidents and risks, raising a question in how two genders differ in the pathogenesis of HCC.

While NAFLD-associated HCC is closely associated with deregulation in fat metabolism, gender dimorphisms may occur in our adipose homeostasis. Differences in body fat composition rather than BMI were suggested to be true determinants of HCC prognosis. Indeed, visceral adiposity can independently predict mortality in patients with HCC (Setiawan et al., 2016). Coincidentally, visceral fat accounts for a strikingly larger proportion of body fat in men than in women, indicating the potential importance of the studies into HCC-associated fat metabolism and the corresponding gender disparities (Fujiwara et al., 2015; Setiawan et al., 2016).

Current studies in the gender disparities in HCC primarily focus on the deregulated signaling pathways and metabolism in hepatocytes. While abnormal fat metabolism plays a crucial role in the onset of HCC, the dramatic differences in the body fat distribution and the adipocyte depot-specific roles in different genders are seldom taken into account. We are presenting the current understanding of the gender disparities in the body fat composition and glycerol metabolism, and discuss the mechanisms by which these metabolic alterations potentially contribute to the contrasting number of incidents of HCC in males and females.

## Adipocyte Metabolism and HCC

Metabolic syndromes are closely associated with two types of white adipose tissues, visceral adipose tissue (VAT) and subcutaneous adipose tissue (SCAT). Gender disparities occur in the distribution and composition of body fat, which may help explain the cause for the strikingly higher HCC incidence in men. VAT differs from SCAT in terms of anatomy, cellular functions, molecular compositions, endocrine functions, and their response of insulin and other hormones. Anatomically, VAT and SCAT are categorized by their corresponding location of depots. Visceral fat is defined as the fat depots around the abdominal organs including the liver and the small intestine; subcutaneous fat, which is located at the femerogluteal regions, back and anterior abdominal wall, represents the physiological buffer for excess energy intake and accounts for around 80% of our total body fat (Wajchenberg, 2000). Visceral fat accumulates as the age increases in both genders (Wajchenberg, 2000). As mentioned, men possess nearly 30% more visceral fat than women, suggesting the promising field to study in resolving the relationship among adipocyte physiology, genders and HCC (Fujiwara et al., 2015; Setiawan et al., 2016). Regarding their particular locations, visceral fat and subcutaneous fat employ different drainage routes of blood. Venous blood from VATs is circulated directly to the liver via the portal vein while subcutaneous fat venous blood is drained through systemic veins (Klein, 2004). Adipocytes are responsible for the storage of excess energy intake and the absorption of free fatty acids (FFAs) and triglycerides. Adipocytes get dysfunctional when they get hypertrophic (Ibrahim, 2010). Adipocytes in VAT are generally bigger in size, hyperlipolytic, insulin-resistant and are molecularly different from those in the subcutaneous regions (Ibrahim, 2010). The accumulation of visceral fat in the omental region promotes insulin resistance and its associated liver injury. The deregulated release of FFAs and glycerol from visceral adipocytes can also contribute to the onset of HCC. Considering the fact that visceral fat accumulates more in males and it causes a tumor-promoting condition in the liver, it is logical to postulate the intriguing relationship between fat metabolism and the gender dimorphisms in HCC.

The adipose tissue comprises multiple cell lineages including adipocytes, preadipocytes, vascular and neural cells and more importantly the immune cells (Ibrahim, 2010; Gregor and Hotamisligil, 2011). Early studies reported the increase in infiltration of inflammatory macrophages in VAT, which thus causes insulin resistance in mice and human (Weisberg et al., 2003; Bruun et al., 2005; Michaud et al., 2012). C-C motif chemokine receptor 2-knockout (Ccr2-KO) mice attributed insulin resistance in diet-induced obesity to the shift of from pro-inflammatory M1 macrophages into alternatively activated M2 macrophages and the reduction of anti-inflammatory cytokine interleukin-10 (IL-10) (Lumeng et al., 2007). The increased infiltration of inflammatory macrophages in visceral fat causes insulin resistance via the nuclear factor-κB (NF-κB) pathway (Lê et al., 2011). In general, visceral fat accumulation is accompanied with the increase in M1 macrophage population, nurturing an elevated local inflammation and a pro-carcinogenic environment within the adipose tissue. The accumulation of activated M2 macrophages in the abdominal adipose tissue remove the protective effect of IL-10 against tumor formation. The increased infiltration could also elevate the liver burden and gradually promotes HCC.

Both levels of visceral fat and HCC risks positively correlate with age and a masculine gender. In view of the harmful effects of visceral adiposity, the contrasting levels of visceral fat accumulation in males and females may help explain the gender disparities in immune responses (Klein and Flanagan, 2016) and HCC development (Tian et al., 2013).

### Visceral Adipose Tissue and HCC

**FIGURE 1:**
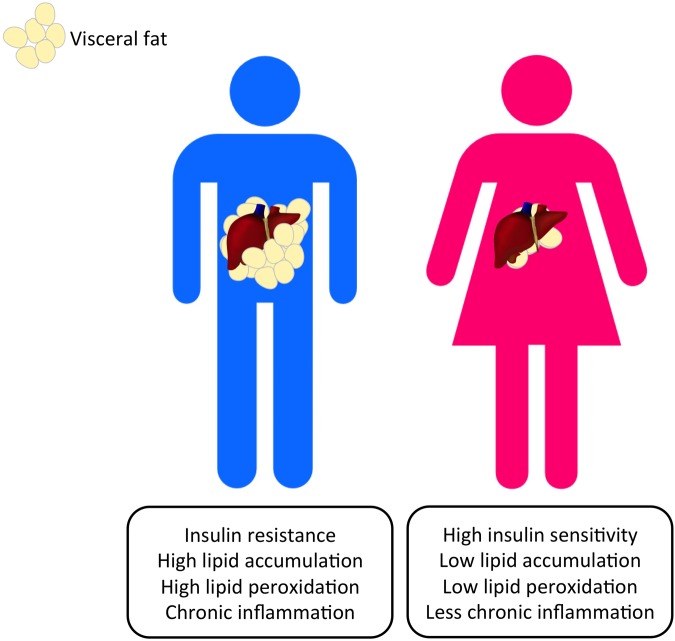
**Gender dimorphism occurs in the altered metabolic pathways that can lead to the progression of HCC.** Visceral adiposity can be observed more commonly in men and nurtures a pro-carcinogenic microenvironment that promotes HCC initiation.

**FIGURE 2:**
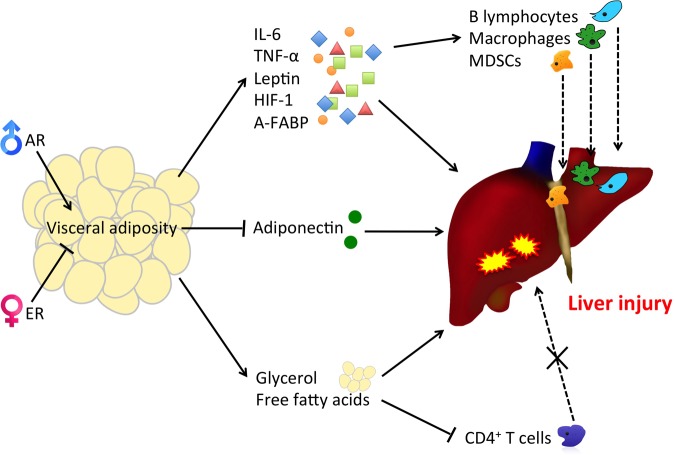
**Visceral adiposity increases the release of pro-inflammatory adipokines and reduces the protective counterpart, causing immune cell infiltration, liver injury, and the subsequent pathological syndromes.** The increase in the release of FFAs and glycerol to the liver from visceral fat also promotes lipid peroxidation and thus increases HCC risks. MDSCs: myeloid-derived suppressor cells.

Obesity is a heterogeneous disorder, with which obese individuals exhibit a wide range of body fat distribution and the associated metabolic profiles. Obesity is associated with excess body fat. Adipose tissue has long been considered as an inert storage of energy while more recent studies have pointed out its role in the immune and endocrine regulation (Cabia et al., 2016). White adipose tissue can be subdivided into VAT and SCAT according to its distribution. VAT mainly deposits around the abdominal viscera in mesentery and omentum and is responsible for the “central obesity” phenomenon in which individuals have a greater risk of type 2 diabetes, cardiovascular diseases and obesity-related cancer (McKeigue et al., 1991; Pérez-Pérez et al., 2009; Ibrahim, 2010; Wolin et al., 2010). VAT and SCAT present very distinct physiological functions. VAT acts as a metabolically active endocrine organ secreting a number of adipokines for regulatory purposes (Ibrahim, 2010). Visceral adiposity has been complicated with negative effects including insulin resistance and the elevated production of pro-inflammatory molecules leptin (LEP), resistin, tumor necrosis factor-α (TNF-α), IL-6, hypoxia-inducible factor 1 (HIF-1) and adipocyte fatty-acid binding protein (A-FABP) (Weisberg et al., 2003; Wisse, 2004; Gregor and Hotamisligil, 2011; Spoto et al., 2014; Petrangeli et al., 2016). Excess absorption of fat from the diet enlarges adipocytes and stimulates the release of FFAs (Ibrahim, 2010) (**Figure 2**). Visceral fat is considered as a major source of FFAs in the portal vein, which can cause liver fat accumulation and the development of NAFLD (Paschos and Paletas, 2009). Adiposity has also been reported to associate with cancer-causing phenotypes including secretion of immune cell-attracting molecules (e.g., monocyte chemoattractant protein 1, MCP-1), angiogenesis, cellular senescence through microbial metabolites (e.g., deoxycholic acid, DCA), lipolysis and decreased production of adiponecitin (Yu et al., 2006; Bruemmer, 2012; Duan et al., 2013; Ferrente, 2013; Yoshimoto et al., 2013) (**Figure 2**). FFAs-induced inflammation and insulin resistance leads to pathogenic endoplasmic reticulum stress and eventually fosters a tumor-promoting, low-grade chronic inflammatory microenvironment in the liver (Ohki et al., 2009; Zhao and Lawless, 2013). A particular form of FFAs, linoleic acid, which is usually accumulated in NAFLD, has recently been ascribed to the disruption of mitochondrial function, oxidative damage and the selective loss of intrahepatic CD4^+^ T cells (Ma et al., 2016). Adiposity is closely associated with the immune cell population in liver. Alongside the chronic inflammation caused by the increase in FFA release, the loss of CD4^+^ T cells potentially results in the failure of recruiting innate immune cells to kill pre-malignant senescent hepatocytes and suggesting the role of an inflammatory microenvironment in HCC initiation. An increased population of B lymphocytes was found recruited in adipose tissue in obese subjects (**Figure 2**). B cells in obese mice favored the secretion of pro-inflammatory cytokines IL-6 and suppressed anti-inflammatory cytokines IL-10 (Winer et al., 2011). High-fat diets raised B cell population and caused insulin resistance in mice (Winer et al., 2011). VAT accumulation has also been proposed as an independent risk factor for HCC since visceral fat accumulation was positively correlated to the recurrence of HCC and the severity of fatty liver in NAFLD patients, but, interestingly, not SCAT (Ohki et al., 2009). Cohort studies using computerized tomography (CT) to measure VAT have shown that visceral fat accumulation enhances the severity of hepatic inflammation and fibrosis in NASH, HCC risks as well as the recurrence of HCC after curative treatment (van der Poorten et al., 2008; Ohki et al., 2009; Vongsuvanh et al., 2012; Schlesinger et al., 2013). Furthermore, visceral fat accounted up to 10–20% of the total fat in men and 5–8% in women, whereas subcutaneous fat accounts for a significantly larger portion of total body fat in women as shown in different studies (Bhaskaran et al., 2014; Setiawan et al., 2016) (**Figure 1**). Emerging evidence in visceral fat accumulation points toward tumor formation and the differential body fat composition in males and females potentially results in gender disparities in HCC risks. The higher level of metabolically active VAT in males nurtures a pro-carcinogenic environment while the higher SCAT level in females may, on the other hand, offers a buffering and protective effect against HCC formation. Regarding the tumor-promoting properties of visceral fat and the coinciding gender dimorphisms in both liver cancer and visceral adiposity, the following session will focus on the underlying mechanisms (**Figure 2**).

In short, the higher level of VAT in males contributes to the likelihood of HCC initiation either by releasing pro-inflammatory adipokines, metabolites or immune cell infiltration.

### Adipokines and HCC

Adipokines are peptides, proteins, and cytokines synthesized by the adipose tissue. Over 75 adipokines have been identified in adipose tissue, while only some of them are associated with carcinogenesis, especially the development of liver cancer (Trayhurn and Wood, 2004; Wood et al., 2009; Zhao and Lawless, 2013; Cabia et al., 2016). Classical examples of adipokines synthesized by dysfunctional obese adipose tissue are usually involved in localized chronic inflammation. Enlarged adipocytes in VAT liberates FFAs to stimulate adipose tissue macrophages to produce TNF-α, which in turns activate adipocytes to undergo lipolysis and secret multiple cancer-causing adipokines (Ibrahim, 2010; Michaud et al., 2012). MCP-1 is secreted by adipocytes to recruit macrophages and myeloid-derived suppressor cells (MDSCs) to local tissue and further worsen chronic inflammation (Yu et al., 2006; Okwan-Duodu et al., 2013) (**Figure 2**). Moreover, hypertrophied adipocytes are exposed to a hypoxic condition in which there is an elevated production of HIF-1, leading to the infiltration of macrophages and the secretion of adipokines (Trayhurn et al., 2008) (**Figure 2**). The release of a pro-inflammatory cytokine IL-6 from hepatic stellate cells is associated with an increased level of FFAs in VAT (Wieckowska et al., 2008; Yoshimoto et al., 2013). IL-6 is also associated with insulin resistance, fibrosis and tumorigenesis involving the activation of STAT3, Akt, Erk, JNK pathways (Wieckowska et al., 2008; Park et al., 2010).

Leptin is an important adipose-derived hormone involved in energy homeostasis, immune responses, angiogenesis, and insulin signaling (Franckhauser et al., 2006). Leptin overexpression was found responsible for TNF-α and IL-6-related chronic inflammation, insulin resistance, liver fibrosis, and HCC development (Ikejima et al., 2001; Wang et al., 2001; Franckhauser et al., 2006; Chen et al., 2007; Tsochatzis et al., 2008). Leptin has been reported to cause proliferation of HCC cells by altering the activity of cyclin D1 and the apoptotic gene, Bax (Chen et al., 2007). Leptin is also one of the reported biomarkers for early recurrence of HCC after treatment (Watanabe et al., 2011). Women require twice as high circulating leptin levels to maintain normal body weight and are more resistant to leptin activity than men do (Licinio et al., 1998). The resistance to leptin in women potentially reduces the corresponding harmful effects. A reduction in leptin sensitivity may reduce cytokine-induced inflammation and insulin resistance. It is suggested that women are subject to a lower HCC risk due to a lower sensitivity to leptin.

Adiponectin is considered to play a protective role in cancer progression involving anti-inflammation, sensitization of insulin signaling and anti-angiogenesis. Adiponectin induces the production of IL-10, working together to inhibit inflammatory factors like IL-6 by inhibiting the NF- κB pathway cascade (Ibrahim, 2010; Lira et al., 2012). Other anti-cancer mechanisms of adiponectin include promoting apoptosis in HCC cells and inhibit cell migration by inhibiting Akt/STAT3 signal transduction (Sharma et al., 2010; Lira et al., 2012). Contradictory results showed the cancer-promoting role of adiponectin in HCC patients. A significantly higher level of serum adiponectin is reported in HCC patients and was also found guilty for tumor growth (Chen et al., 2012; Sadik et al., 2012). Since the metabolic profiles in HCC patients have been severely altered, the implicit role of adiponectin regarding HCC development requires further investigations. Despite all these, it is generally believed that men with a higher level of visceral fat accumulation are more subject to a procarcinogenic microenvironment. Hormonal influences on adipocyte homeostasis could be one of the reasons for the differential visceral fat accumulation and the differences in adipokine production.

## Gender Dimorphism In Adipocyte Metabolism

### Sex Hormones and Adipocyte Metabolism

Visceral fat deposition is significantly higher in males than in females whereas subcutaneous fat accumulates more in females (Ibrahim, 2010). Having a similar total fat and BMI, males showed a higher visceral fat and liver fat content than females in multiple datasets (Bhaskaran et al., 2014; Setiawan et al., 2016). Obvious gender dimorphism was also observed in both diet-related liver diseases and HCC initiation (Chang et al., 2016). Visceral fat actively secrets oncogenic adipokines, leading to chronic inflammation and contributes to liver cancer. The disparities of liver cancer and visceral fat deposition potentially originate from high androgen receptor (AR) density (Freedland, 2004). The increase in body visceral fat and the decrease in body subcutaneous fat as the age increases, coinciding with the increase with HCC incidence (Björntorp, 1995). Estrogen promotes the accumulation of subcutaneous fat and is protective against inflammation (Ibrahim, 2010). After menopause, the rise of body VAT in women is related to the deficiency of estrogen (Björntorp, 1995; Freedland, 2004). Signaling pathways involving sex steroids contribute to the differential secretion of cancer-causing adipokines in men and women, thus leading to a higher risk of HCC in men. Potential sexual dimorphism in HCC is also caused by the differential recruitment of Foxa-1/2 transcription factors and the corresponding androgen and estrogen receptors, showing the involvement of steroid hormones in liver cancer development (Li et al., 2012). AR and ER can both act as transcription factors altering chromatin accessibility and gene transcription in HCC (Li et al., 2012). Although limited studies using chromatin-immunoprecipitation-sequencing (ChIP-seq) have been done on adipocytes, it is postulated that the differential binding profiles of AR and ER in visceral fat and subcutaneous fat can help unveil the underlying mechanisms of androgen and estrogen in visceral adiposity and the subsequent side effects. The potential links may help understand the differences of adipokine profiles in men and women and thus explain how men get more prone to HCC initiation.

#### Estrogen

Estrogen and estrogen receptor (ER) signaling have been found to have a protective role in HCC initiation and progression via the IL-6/STAT inflammatory pathways (Naugler et al., 2007; Yang et al., 2012). One of the anticancer adipokines adiponectin is expressed at a higher level in women than in men (Laughlin et al., 2006). Estrogen replacement therapy helps decrease adiponectin circulation in postmenopausal women (Kunnari et al., 2008). It is reported that adiponectin is inversely associated with the incidence of multiple types of diet-related cancer (Housa et al., 2006). Adiponectin is associated with anti-inflammation, increasing insulin sensitivity and anti-angiogenesis, which coincides with the protectively role of estrogen signaling (Tworoger et al., 2007; Ibrahim, 2010). A reduction of body estrogen after menopause is frequently followed by an increase in body visceral fat (increase in size of adipocytes), hyperinsulinemia and increase in IL-6 production (Freedland, 2004). Estrogen offers a protective effect against metabolic deregulation and HCC. It is suggested that a reduction in the endogenous estrogen level causes a drop in adiponectin in postmenstrual women. The physiological mechanisms of estrogen on the release of adiponectin are a potential explanation for the gender dimorphism of HCC.

#### Androgen

Regional androgen concentration in VAT suggests a depot-specific effect on adipocyte functions and metabolism (Bélanger et al., 2006). Androgen excess in female mice using 5α-dihydrotestosterone (DHT) treatment induced an increase in visceral fat deposition, via an altered hypothalamic axis (Nohara et al., 2014). The complicated role of androgen signaling in body fat distribution and adipocyte functioning has long annoyed scientists for decades. Early studies revealed results on a reduced circulating androgen levels with visceral fat deposition (Garaulet et al., 2000; Tsai et al., 2004; Kraus et al., 2015). Androgen was shown to have a suppressive effect in adiponectin level consistently in cell lines, animal models, and human epidemiological studies (Nishizawa et al., 2002; Lanfranco et al., 2004; Isobe et al., 2005; Page et al., 2005). Such phenomenon explains how androgen undermines the anti-inflammatory effects of adiponectin and develops chronic inflammation in the liver. Regarding the regulation of androgen on genes involved in adipogenic genes, recent studies have discovered the role of microRNA miR-375 in adipocyte differentiation and distribution. Androgen treatment in preadipocytes could downregulate miR-375 expression and increase the level of adiponectin receptor 2 (ADIPOR2), which might help understand the mechanisms of how testosterone deficiency could lead to insulin resistance and visceral fat accumulation (Kraus et al., 2015). Generally, visceral fat has a high density of AR, which allows testosterone to amplify its own effect by raising AR expression, inhibiting lipoprotein lipase and the uptake of FFAs (Wajchenberg, 2000; Freedland, 2004). As men age, the bioavailability of testosterone declines and leads to the deposition of visceral fat. Yet, the case is opposite in women who exhibit an increase in androgen level released from the ovary when they are complicated with hyperinsulinemia and it is known as the polycystic ovary syndrome (Wajchenberg, 2000; Freedland, 2004). *In vivo* and *in vitro* studies of leptin production varied greatly. Adipocytes from different locations reacted differently to androgen *in vivo* and *in vitro* (Machinal et al., 1999). The difficulty of local application of androgen has long limited the physiological studies of how androgen affects a particular region of adipocytes and the following pathologies. Contrary to its involvement in HCC, androgen appears to inhibit visceral fat accumulation and the complicated association among androgen, visceral fat accumulation and liver cancer needs further investigation.

In short, the androgen to estrogen ratio determines the leptin and adiponectin levels and visceral adiposity. A higher androgen to estrogen ratio is associated with a respective higher leptin and lower adiponectin ratio in both genders. The high androgen to estrogen ratio is linked to metabolic syndromes and it is also predicted such phenomenon is one of the culprits for HCC initiation.

### Gender-specific Single Nucleotide Polymorphisms in Adipose Tissue

**FIGURE 3:**
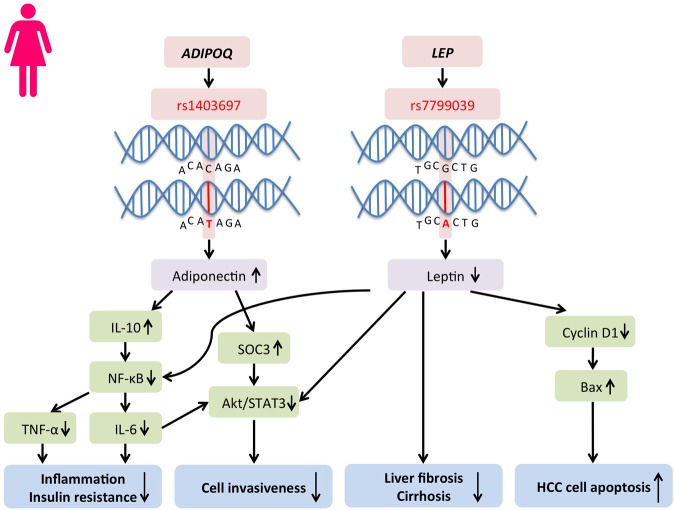
**Protective single nucleotide polymorphisms (SNPs) in ADIPOQ and LEP genes that lead to differential serum adiponectin and leptin levels occur frequently in females.** A respective higher adiponectin and a lower leptin level can be found in female subjects. SNPs in ADIPOQ and LEP genes potentially reduce HCC risks and development in women through inflammatory and oncogenic pathways.

The large discrepancies in fat distribution and homeostasis in males and females imply potential genetic predispositions. Single nucleotide polymorphisms (SNPs) are enriched for expression of loci of quantitative traits and may alter gene expression (ENCODE Project Consortium, 2012). This mechanism has been argued to be a major driver in disease susceptibility by genetically altering the susceptibility of DNA methylation and thus affecting gene expression (Martínez et al., 2014). It has been demonstrated for obesity, insulin resistance, inflammation, oxidative stress, and hypoxia, which are all driver components of HCC initiation (Martínez et al., 2014). Such mechanism is also reported to be cell type-specific (Fairfax et al., 2012). Sex-specific SNPs are found potentially responsible for fat distribution and inflammation (Sung et al., 2016; Wang et al., 2016). Martínez et al. (2014) have summarized a panel of candidate genes which are epigenetically regulated through DNA methylation and are involved in lipid homeostasis, including *CEBPA, PPARA, LEP, MC4R, NPY, POMC, FTO, ADIPOQ, GLUT4, INS, HIF1A, IFNG, TNF, FASN, NR3C1, and UCP1* (Martínez et al., 2014). Among all the epigenetic targets, sexual dimorphisms in the SNPs can be identified, including the Adiponectin, C1Q And Collagen Domain Containing gene (*ADIPOQ)* and the leptin gene *(LEP)*, which, respectively, encode for crucial players in visceral adiposity and liver cancer initiation, adiponectin and leptin (**Figure 3**).

#### ADIPOQ

*ADIPOQ* has a length of 1.579 kb and contains three exons, in which the transcription start site is located at exon 2 (Mackevics et al., 2006). Several SNPs inside the *ADIPOQ* gene have been associated with adiponectin serum levels, body adiposity and other metabolic alterations, rendering it a potential player in obesity and metabolic syndromes (Mackevics et al., 2006; Dolley et al., 2008; Bostrom et al., 2009). Association studies of *ADIPOQ* polymorphisms, adiponectin levels and obesity phenotypes using samples of African American population from the Jackson Heart Study (JHS) cohort (Riestra et al., 2015). Data collected from 2968 participants (1131men and 1837 women) revealed gender-specific association between SNPs rs6444174, rs1403697, and rs7641507 with serum adiponectin levels in women but not men (Riestra et al., 2015). SNPs in *ADIPOQ* have also been reported to be associated with type 2 diabetes (Peters et al., 2013). SNPs at the adiponectin gene appear differently in men and women affecting circulating adiponectin, which may cause different adipocyte homeostasis and thus affecting the liver functions in a sex-specific manner.

#### Leptin (LEP)

**FIGURE 4:**
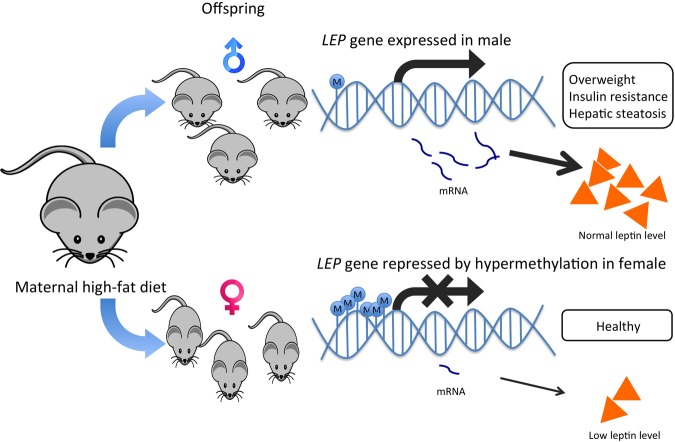
**Hypermethylation of *LEP* promoter in female offspring offers a protective effect against maternal high-fat diets by lowering leptin production in mice.** Maternal high-fat diets affect offspring’s epigenetic regulation on the *LEP* gene. Obesogenic environments during the pre-conceptional period and the early phase of development led to a lower methylation state of the *LEP* promoter in male rodent offspring than their female counterparts. The higher leptin production in male offspring was also associated with overweight, insulin resistance and hepatic steatosis, which could help explain the gender dimorphism in leptin production and the development of liver diseases and HCC.

DNA polymorphism in the leptin gene is associated with tumorigenesis of multiple cancer types, such as lung cancer, lymphoma, thyroid cancer, and colon cancer (Slattery et al., 2008; Liu et al., 2014; Unsal et al., 2014; Marcello et al., 2015). *LEP* is primarily expressed in differentiated adipocytes of white adipose tissue, and the hormone leptin it encodes for, plays a role in the regulation of food intake and the expression of energy-regulating peptides. *LEP* has been proposed as a culprit for obesity-induced cancer because it displays epigenetic variation and is involved in energy homeostasis, immune responses, angiogenesis and insulin signaling (Franckhauser et al., 2006). Deregulated production of leptin or its receptor is highly associated with HCC development (Chen et al., 2007). Some Leptin G-2548A (rs7799039) polymorphism showed association with serum leptin level and obesity in young females only (Ben Ali et al., 2009; Shahid et al., 2015) (**Figure 3**). Since leptin is closely related to insulin signaling and inflammation, it is considered as a strong risk factor in obesity-related cancer. The gender dimorphism in the leptin gene may alter leptin secretion in adipocytes and eventually alter the cancer risk in different genders (Tobi et al., 2009). On the other hand, when the preadipocytes mature, the gene is activated by DNA demethylation (Melzner et al., 2002). Differences in the methylation status of the *LEP* promoter influence *LEP* expression *in vitro*, which approves the functional role of DNA methylation on leptin secretion in adipocytes (Rankinen et al., 2006). The establishment of epigenetic profiles is susceptible to maternal diets during early development (Dahlhoff et al., 2014). Female mice undertaking high-fat diets affected the offspring genes involved in fatty liver disease, lipid droplet size regulation and body fat expansion (Dahlhoff et al., 2014). The harmful effects of maternal diets on offspring have been reported to be organ-specific, gender-specific and remain at different life stages. The adult male offspring exhibited traits related to liver disease development overweight, insulin resistance, high leptin levels and hepatic steatosis but not the female counterpart, proving the possibility of gender-specific expression of leptin (Dahlhoff et al., 2014; Ge et al., 2014; Allard et al., 2015) (**Figure 4**).

Single nucleotide polymorphisms in the genes controlling expression of key adipokines leptin and adiponectin exert differential effects on males and females. Metabolic syndromes including type 2 diabetes and excess fat deposition could be caused by the differential expressions of *ADIPOQ* and *LEP* genes in males and females as a result of the respective SNPs.

GWAS studies rely heavily on the associations of gender-specific SNPs with metabolic traits such as insulin, leptin, adiponectin levels to support the fact that certain genetic predispositions leading to visceral fat accumulation and the release of leptin increases the risks of metabolic complications. The following challenge now is to understand the biological functions of those genes and how they are regulated differentially in males and females. In the aspect of genetics, mRNA expression profiles of such genes can be correlated with BMI, body fat distribution and our gender. Other possible mechanisms include epigenetic regulation via DNA methylation or histone modifications may be responsible for the association of the aforementioned traits with gender.

### Epigenetic Regulation in Adipose Tissue

Epigenetic regulation of genes are considered to be important for disease pathogenesis since it is believed to translate environmental factors into phenotypic traits through alteration in the transcriptome (Fraga et al., 2005; Sandovici et al., 2011).

#### DNA Methylation

One of the many epigenetic mechanisms, methylation of CpG dinucleotides is proved to play crucial roles in the decision of cell fates, tumorigenesis and multiple cellular functions (Esteller, 2007; Baylin and Jones, 2011). DNA methylation usually occurs at CpG-rich regions and is often associated with gene repression (Bird, 2002). Epigenetics can provide insights for the understanding of chronic disease onset in adults, which interact with external stimuli like dietary intake and nutritional processes. Epigenetic regulations are believed to translate physiological impacts into altered gene expressions. Many genomic studies have reported the gene expression patterns in VAT, yet the differentially expressed genes may be functionally associated with visceral fat dysfunction and metabolic syndromes, including adipose tissue macrophage-specific genes and insulin resistance-related genes (Bouchard et al., 2007; Blüher, 2009; Hardo et al., 2011; Klimcakova et al., 2011). Epigenetic mechanisms may contribute to the variability of the gene expression and the different components of metabolic syndromes. Transcriptional CpG-island promoter hypermethylation or global DNA methylation may affect the expression of genes controlling adipokine secretion, fat distribution, macrophage infiltration and insulin sensitivity. A recent genome-wide analysis of CpG methylation states of blood and adipose tissue from 479 individuals has revealed the association between DNA methylation and BMI (Dick et al., 2014). Data showed that the methylation level at three *HIF3A* sites was strongly correlated to BMI in adipose tissue, i.e., an increase in BMI was associated with an increase in methylation level at *HIF3A* sites. The HIF3A gene could not only respond to oxygen content locally, but it also played a role in cellular response to insulin and glucose and accelerated adipocyte differentiation in acquired obesity (Heidbreder et al., 2007; Hatanaka et al., 2009; Robciuc et al., 2011). Obesity has been reported to increase a global mean DNA methylation in adipocytes (Arner et al., 2015; Benton et al., 2015). Exercise can significantly influence the DNA methylation patterns in a genome-wide manner (Rönn et al., 2013). Post-obese patients experienced a global DNA hypomethylation and differential methylation of adipogenesis genes (Dahlman et al., 2015). More DNA methylation in adipocytes can regulate different genes related to hypoxia and insulin sensitivity, providing a new scope for investigating the relationship between adipocyte homeostasis and liver cancer development.

Recent studies have also take advantage of cell lines and human mesenchymal stem cells in the study of DNA methylation in the transcriptional activation of leptin and adiponectin genes (Kuroda et al., 2016; Zhang W. et al., 2016). On top of this, Ambati et al. (2016) have also demonstrated the differential expressions of genes encoding for epigenetic regulators in VAT and SCAT in mice. The adipocyte nuclei were immuno-captured from VAT and SCAT to resolve the difficulties of isolating mature adipocytes from adipose tissue. The differential expressions of chromatin remodeler proteins involved in DNA methylation and histone modification shed light on the studies of epigenetic regulation of gene expressions in adipose tissues, promoting the study in the gender disparities in adipocyte homeostasis through epigenetics. Possible mechanisms of such disparities may include the binding of transcription factors like AR and ER alongside other pioneer factors that affect the chromatin status (Iwafuchi-Doi et al., 2016).

#### Histone Modifications

Histone modifications represent another important mechanism of epigenetic regulation (Baylin and Jones, 2011). Histone acetyltransferases (HATs) and histone deacetylases (HDACs) have been shown to respond to regulatory signals involved in adipocyte differentiation and adipogenesis (Zhou et al., 2014). HDAC9 negatively regulated adipogenic differentiation in HDAC9 overexpression and knockout experiments (Chatterjee et al., 2011). During chronic high-fat feeding in mice, HDAC9 deletion was shown to offer a protective effect against metabolic diseases by increasing adiponectin expression (Chatterjee et al., 2014). Histone deacetylase HDAC4 is repressed in exercise-induced DNA methylation, leading to a reduced repressive activity on glucose transporter GLUT4 and thus increasing adipocyte glucose uptake and lipogeneis (Rönn et al., 2013). Recent studies have also shown the effect of H3K9 methylation at Cebpa and Pparg loci marked with H3K4me3 deposition limits CCATT/enhancer binding protein β binding and thus halting adipogenesis (Matsumura et al., 2015). Although very limited studies have been done on histone modification profiling in adipocytes, more and more evidence is pointing toward the roles of histone modifiers in adipocyte functions the pathological development of metabolic diseases. Epigenetic studies of adipocyte metabolism may shed light on the disease progression as well as the gender differences in metabolic diseases and HCC.

### Gender Dimorphism in MiRNAs Actions in Adipocyte Tissue

MicroRNAs (miRNAs) are small noncoding RNAs of 18–15 nucleotides that regulate the translation of messenger RNAs (mRNAs) through binding to their 3′-untranslated regions (UTRs) leading to translation inhibition and/or mRNA degradation (Wong et al., 2008). miRNAs are known to be involved in multiple biological functions, including cell proliferation, cell differentiation, metabolism, and immunity and in diseases such as cancer, cardiovascular diseases and type 2 diabetes (Mendell, 2005). In adipocytes, the expression of miRNAs and the corresponding regulation are closely related to sex hormones androgen and estrogen, providing a promising scope to understand gender dimorphism in lipid metabolism.

#### miR-125a/b

The miR-125 family comprises miR-125a and miR-125b with similar sequences. The miR-125 family has been reported to play a role in the initiation and progression of cancers by either acting as tumor suppressors or oncogenes (Cowden Dahl et al., 2009; Jiang et al., 2010). However, it is clear that miR-125a down-regulation is clear linked to the development of leukemia (Ufkin et al., 2014). MiR-125a is also found responsible in adipose tissue and immune development (Sun et al., 2009; Guo et al., 2010). MiR-125a targets and inhibits the expression of estrogen-related receptor alpha (ERRα), a nuclear receptor that plays an important role in a number of metabolic homeostasis processes, such as fatty acid metabolism (Huss et al., 2004; Ji et al., 2014). It was also found that miR-125a is overexpressed in diabetic rats as compared with non-diabetic ones (Herrera et al., 2009). Intriguingly, the overexpression of miR-125a has been found to inhibit adipocyte differentiation and the underlying gender-specific mechanisms of how miR-125a works together with ERRα requires further investigation (Herrera et al., 2009). Although the relationship of miR-125 and estrogen signaling in adipocytes has not been well established, estrogen is found to alter miR-125 family in other cell types. MiR-125b can be up-regulated by estrogen via ERα pathway, which thus protects against NAFLD in mouse hepatocytes by inhibiting fatty acid synthesis and preventing lipid accumulation in liver (Zhang et al., 2015) (**Table 1**).

**Table 1:** Target genes, functions of sex hormone-associated miRNAs in adipocytes.

miRNA	Expression in Obesity	Expression in T2D	Targets	Upstream	Functions	Reference
miR-125a	Up	Up	Inhibit ERRα	?	Inhibit adipocyte differentiation; Inhibit ERRα; Reduce insulin sensitivity	Huss et al., 2004; Herrera et al., 2009; Sun et al., 2009; Guo et al., 2010; Ji et al., 2014; Yeh et al., 2014
miR-125b	Down	Down	?	Up-regulated by ERα	Inhibit NAFLD; Inhibit lipid accumulation; Inhibit fatty acid synthesis	Xie et al., 2009; Ortega et al., 2010; Zhang et al., 2015
miR-222	Up	Up	Inhibit ERα, GLUT4	Up-regulated by high glucose and estradiol	Reduce insulin sensitivity	Campbell and Febbraio, 2002; Livingstone and Collison, 2002; Xie et al., 2009; Herrera et al., 2010; Retnakaran et al., 2010; Rao et al., 2011; Shi et al., 2014; Deiuliis, 2016
miR-301a	?	Up	Inhibit AR	?	Promote metastasis; Increase TGF-β1 expression	Derynck et al., 1998; Qi et al., 2008; Panguluri et al., 2013; Xie et al., 2015
miR-375	Down	Down	Inhibit ADIPOR2	Induced by ERK-PPARg-aP2 signaling; Inhibited by androgen	Promote fat accumulation; Reduce insulin sensitivity	Yamauchi et al., 2003; Krek et al., 2005; Bauer et al., 2010; Ling et al., 2011; Kraus et al., 2015


#### miR-222

In a study of 3T3-L1 adipocytes, miR-222 was found upregulated when exposed to a high extracellular glucose concentration and in obese adipocytes (Xie et al., 2009; Herrera et al., 2010). High estradiol concentrations in visceral adipocytes were reported to increase miR-222 expression and decrease ERα and GLUT4 expression (Shi et al., 2014). Previous findings have also demonstrated the role of estrogen, androgen, and other hormones in insulin sensitivity (Livingstone and Collison, 2002; Retnakaran et al., 2010). High estrogen level can reduce insulin sensitivity by mediating ERα and ERβ (Barros et al., 2009). *In vivo* and *in vitro* studies indicated that a high concentration of estrogen could inhibit the expression of the insulin-sensitive transporter GLUT4 in adipose tissue, muscle, and liver (Campbell and Febbraio, 2002). ERα was also found to be a direct target of miR-222 in breast cancer cells, with a specific binding site at the seed sequence of miR-222 (Rao et al., 2011). miR-222 could be an important regulator of ERα expression in insulin resistance. However, a clearer pathway needs to be elucidated by further overexpression of miR-222 to determine whether it directly acts on ERα and GLUT4 expression (Shi et al., 2014). Possible signaling pathways of the action of miR-222 includes β-catenin and TGF-β signaling (Rao et al., 2011) (**Table 1**).

#### miR-301a

Preadipocytes can proliferate and differentiate into an adipose deposit upon stimulation and leads to an increase in adipocyte numbers and leading to obesity (Rosen and MacDougald, 2006). Although the relationship between preadipocyte infiltration and the initiation of HCC remains unclear, studies have shown that the recruitment of preadipocytes is responsible for enhancing prostate cancer invasion and breast cancer development (Rama-Esendagli et al., 2014; Xie et al., 2015). Preadipocytes were found to induce miR-301a to promote metastasis of prostate cancer by down-regulating AR (Xie et al., 2015). Down-regulation of AR could regulate translationally and increase TGF-β1 expression (Qi et al., 2008). Following the Smad signaling mediators, the down-regulation of AR consequentially activated target genes like matrix metallopeptidase 9 (MMP-9) and promote tumor metastasis (Derynck et al., 1998; Wang et al., 2013). Although AR overexpression was present in progressive HCC, AR was also found to be essential in suppressing HCC metastasis in the later stage of tumor development (Zender and Kubicka, 2008; Ma et al., 2012). A high AR expression could reduce the phosphorylation and inhibit the activity of p38, NF-κB signaling and MMP-9 expression and the suppress HCC metastasis (Tian et al., 2015). The coinciding evidence of miR-301a expression and AR signaling in preadipocytes in different cancer types may help understand the gender disparity of late-stage HCC progression (**Table 1**).

#### miR-375

The direct binding of miR-375 was mentioned to locate at the 3′-UTR of murine and human ADIPOR2 (Krek et al., 2005; Kraus et al., 2015). ADIPOR2, as a receptor for adiponectin, mediates to increased PPARα ligand activities and fatty-acid oxidation by regulating adiponectin and ADIPOR2 signaling in adipose tissue (Yamauchi et al., 2003; Bauer et al., 2010). The function of miR-375 was reported to be induced during adipogenesis and to promote adipocyte differentiation by suppressing ERK1/2 phosphorylation via the ERK-PPARg-aP2 signaling (Ling et al., 2011). Insulin resistance in adipocytes was associated with a low adiponectin receptor level (Bauer et al., 2010). Increased expression levels of both ADIPOQ and ADIPOR2 could help prevent fat accumulation in liver and adipose tissue and led to increased insulin sensitivity (Ma and Liu, 2013). Studies also showed that the expressions of adipogenesis-promoting miR-375 and ADIPOR2 were decreased and increased, respectively, in androgen treatment (Kraus et al., 2015). Inhibiting miR-375 could decrease adipocyte differentiation and increase ADIPOR2 protein expression (Kraus et al., 2015). From the observations in separate studies, mR-375 may be responsible for the deterioration of insulin-resistance-related liver injury and may cause the difference in the visceral fat deposition and liver diseases in men and women when the body androgen level decreases with age (Bassil et al., 2009) (**Table 1**).

Emerging epigenetic studies in adipocytes reveal a range of regulatory mechanisms in adipogenesis, adipokine expression and other metabolic pathways. Although limited studies have been done in the epigenetic regulations in adipocytes to demonstrate gender dimorphisms, AR and ER have already been shown to alter the epigenetic profiles as a nuclear factor in HCC cells (Li et al., 2012). Our body fat composition may as well be regulated epigenetically and lead to such gender disparities. The epigenetic mechanisms in adipocytes may provide insights in HCC development through metabolic deregulation. With the advancement of the nuclei capture technique in VAT and SCAT mentioned by Ambati et al. (2016) it will be promising to study the depot-specific and even gender-specific epigenetic profiles in adipocytes to unveil the underlying mechanisms in adipocyte homeostasis and its potential effects on HCC initiation.

## Genders, Aquaglyceroporins and HCC

Glycerol-3-phosphate (G3P), which is also known as glycerol, is an important metabolite for the control of fat accumulation and is the responsible for triacylglycerols (TAGs) synthesis and glucose homeostasis. Circulating free glycerol results from lipolysis, diets or in the kidney reabsorption (Reshef et al., 2003). Aquaglyceroporins (AQPs) are protein channels for the transport of glycerol, other small neutral solutes and water across adipocytes. AQPs are emerging as important players adipocyte homeostasis implicated with adiposity and the control of insulin resistance (Rojek et al., 2007). Aquaglyceroporins are sub-classified into AQP3, 7, 9, and 10 which regulate transport of glycerol and insulin sensitivity in adipocytes (Frühbeck, 2005). In line with the gender dimorphisms in glucose and fat metabolism, the different expressions of AQPs and glycerol transport in men and women are also observed in diet-induced diseases including insulin resistance and NAFLD (Rodríguez et al., 2006; Rojek et al., 2007).

Regarding the relationship between AQPs and HCC, emerging evidence has also been ascribing AQP deregulation in hepatocytes to the pathogenesis and, as well, the metastasis of NAFLD-associated HCC. AQP9 has been identified as an important glycerol transport pathway in hepatocytes to facilitate hepatic gluconeogenesis and TAG synthesis (Rodríguez et al., 2011a). AQP9 is the main aquaglyceroporin in the liver and is dramatically reduced in HCC and is localized at non-tumorigenic liver tissue (Padma et al., 2009; Ribatti et al., 2014; Chen et al., 2016). AQP9 knockdown in mice significantly alleviated NAFLD-related symptoms including intrahepatic lipid accumulation and high serum lipid level, possibly due to a decrease in glycerol import into hepatocytes thus reducing liver lipid accumulation (Cai et al., 2013). The results are controversial since the resulted AQP9 knockdown is not tissue-specific and a global effect of AQP9 is observed, including glycerol absorption. However, AQP9 down-regulation was found frequent in hepatic biopsies of obese patients with type 2 diabetes (Catalán et al., 2008). A reduced AQP9 expression in HCC cells was shown to resist apoptosis and the respective overexpression could restore the cell responsiveness to apoptotic stimuli (Jablonski et al., 2007). Protein kinase A activator, dibutyrul cAMP, increased AQP9 expression in HCC cells and suppressed tumor growth *in vivo*, suggesting the important roles of hepatocyte AQP9 in HCC development (Peng et al., 2016). Experiments in the SMMC7721 cell line demonstrated the molecular mechanism of AQP overexpression on its tumor suppressive function. An increase in AQP9 expression repressed the PI3K/Akt pathway and thus increased forkhead box protein O1 expression and up-regulation of apoptotic caspase-3 (Li et al., 2016; Zhang Q. et al., 2016). AQP9 downregulation also promoted HCC metastasis in mice (Zhang Q. et al., 2016). The observed down-regulation of AQP9 in hepatocytes in obesity, diabetes and HCC is believed to be a negative feedback response of high glycerol intake. AQP9 is important in controlling glycerol import in hepatocytes. A proper glycerol import is crucial in maintaining the gluconeogenic pathway in hepatocytes and a normal lipid metabolism in the surrounding adipose tissues. The up-regulation hepatic AQP9 expression was associated with the alleviation the NAFLD-associated symptoms (Rodríguez et al., 2015b). AQP9 in hepatocytes is considered to offer a protective effect on HCC initiation and metastasis. On top of the clear relationship between hepatocyte AQPs with HCC, the interactions between adipocytes and hepatocytes are crucial in studying NAFLD-related HCC development since the distinguishing difference in fat deposition in males and females also affect glycerol transport and the corresponding subsequences. The coinciding gender dimorphism in fat metabolism and HCC development show the prospect in the interrelation between adipocyte and hepatocyte homeostasis.

In adipocytes, AQP7 acts as the main pathway in facilitating release of glycerol, while the others, including AQP3, 10 and 11 contribute glycerol export to a lower extent (Rodríguez et al., 2011a; Laforenza et al., 2013; Madeira et al., 2014). The expression of AQPs also appears to be location-specific. Visceral adipocytes exhibit a higher expression level of AQP3 and AQP7, which may result in the overall rise in the lipolytic rate and glycerol release from VAT (Miranda et al., 2010; Rodríguez et al., 2015a). In contrast, subcutaneous fat with a lower AQP7 level may help promote glycerol accumulation and thus adipocyte hypertrophy (Rodríguez et al., 2015a). AQP regulation in adipocytes is crucial in maintain the fat and glucose homeostasis, i.e., a deregulation will lead to obesity and insulin resistance (Kuriyama et al., 2002; Hibuse et al., 2005).

A higher plasma glycerol level can be observed in females, which can be explained by a higher body fat rate resulting in a faster glycerol turnover (Hedrington and Davis, 2015). Moreover, adipose tissues respond differently to adipokines, lipolytic hormones and probably sex hormones in men and women (Schmidt et al., 2014). Fasting studies and exercise studies have shown that women exhibit a significantly higher glycerol level than men, which may be ascribed to the differences in sensitivity to insulin, epinephrine, leptin and even neural activities (Clore et al., 1989; Davis et al., 2000; Frühbeck et al., 2001, 2014; Hedrington and Davis, 2015). Obesity is associated with a higher lipolytic rate, plasma FFAs and glycerol (Rodríguez et al., 2015a). A gender-specific effect of regular exercise has shown a higher expression of omental adipose AQP7 in females (Rodríguez et al., 2015a).

Besides differential AQP expression, gender dimorphisms also occur in the response to leptin. The adipokine leptin has a lipolytic activity and has been shown to repress AQP7 and AQP9 expression via the PI3K/Akt/mTOR signaling pathway in human adipocytes (Rodríguez et al., 2011b). The association between a higher expression of AQP7 in females and the leptin level requires further investigation (Sjöholm et al., 2005; Dahlhoff et al., 2014). Leptin stimulates translocation of AQP7 to the cytoplasm to facilitate glycerol release from adipocytes (Rodríguez et al., 2015b). At the same time leptin can also downregulates AQP7 mRNA to prevent depletion of fat stores (Rodríguez et al., 2015b). Long-term leptin administration in male leptin-deficit mice could downregulate AQP3 and AQP7 could help alleviate lipid accumulation in adipose tissue and liver (Rodríguez et al., 2011b, 2015b). However, the miscellaneous functions of leptin contradict in the development of obesity and liver cancer (Rodríguez et al., 2015b). The rescuing effect of leptin in NAFLD in leptin-deficient obese mice could be due to the neuroactive effects of leptin, but not as adipokines promoting local inflammation around internal organs. AQP7 is downregulated in women with severe obesity and this trait is limited to obesity but not type 2 diabetes (Ceperuelo-Mallafré et al., 2007). The downregulation of AQP7 was also reported to increase susceptibility of obesity by promoting lipid accumulation in the adipose tissue (Marrades et al., 2006). The complicated association between NAFLD and AQPs involve not only adipocyte glycerol transport, but also the glucose and glycerol transport in hepatocytes. Mechanistic delineation of leptin in adipocytes is required to elucidate its relationship with liver cancer initiation.

## Concluding Remarks

Deregulated energy homeostasis and metabolic disorders induced by obesity and type 2 diabetes are key but largely unresolved issues in HCC development. The alterations in genetic, epigenetic and miRNA control of lipid and glucose metabolism, oxidative stress and the production of pro-inflammatory and anti-inflammatory cytokines contribute to the multiple-step development of NAFLD, NASH, cirrhosis and eventually HCC. The increased incidence of HCC associated with metabolic syndrome is significantly associated with the male gender. Sexual dimorphism as exemplified by the androgen and estrogen signaling pathways shapes the fat distribution in the body, contributing to the differential exposure to pro-inflammatory cytokines and lipotoxic adipokines which influence hepatic malignant transformation. Further mechanistic delineation of adipocyte metabolism via sex hormones using systems approach will shed light on the multifaceted roles of aberrant fat accumulation in HCC initiation and progression.

## Author Contributions

All authors listed have made substantial, direct and intellectual contribution to the work, and approved it for publication.

## Conflict of Interest Statement

The authors declare that the research was conducted in the absence of any commercial or financial relationships that could be construed as a potential conflict of interest.

## References

[B1] Allard C. Desgagné V. Patenaude J. Lacroix M. Guilemette L. Battista M. C. (2015). Mendelian randomization supports causality between maternal hyperglycemia and epigenetic regulation of leptin gene in newborns. *Epigenetics* 10 342–351. 10.1080/15592294.2015.1029700 25800063 PMC4622547

[B2] Ambati S. Yu P. Mckinney E. C. Kandasamy M. K. Hartzell D. Baile C. A. (2016). Adipocyte nuclei captured from VAT and SAT. *BMC Obes.* 3:35 10.1186/s40608-016-0112-6 PMC4949929 27462403

[B3] Arner P. Sinha I. Thorell A. Rydén M. Dahlman-Wright K. Dahlman I. (2015). The epigenetic signature of subcutaneous fat cells is linked to altered expression of genes implicated in lipid metabolism in obese women. *Clin. Epigen.* 7:93 10.1186/s13148-015-0126-9 PMC4562340 26351548

[B4] Baffy G. Brunt E. M. Caldwell S. H. (2012). Hepatocellular carcinoma in non-alcoholic fatty liver disease: an emerging menace. *J. Hepaotol.* 56 1384–1391. 10.1016/j.jhep.2011.10.027 22326465

[B5] Barros R. P. Gabbi C. Morani A. Warner M. Gustafsson J. A. (2009). Participation of ERα and ERβ in glucose homeostasis in skeletal muscle and white adipose tissue. *Am. J. Physiol. Endocrinol. Metab.* 297 E124–E133. 10.1152/ajpendo.00189.2009 19366879

[B6] Bassil M. Alkaade S. Morley J. E. (2009). The benefits and risks of testosterone replacement therapy: a review. *Ther. Clin. Risk Manag.* 5 427–448. 19707253 10.2147/tcrm.s3025 PMC2701485

[B7] Bauer S. Weigert J. Neumeier M. Wanninger J. Schäffler A. Luchner A. (2010). Low-abundant adiponectin receptors in visceral adipose tissue of humans and rats are further reduced in diabetic animals. *Arch. Med. Res.* 41 75–82. 10.1016/j.arcmed.2010.02.010 20470935

[B8] Baylin S. B. Jones P. A. (2011). A decade of exploring the cancer epigenome-biological and translational implications. *Nat. Rev. Cancer* 11 726–734. 10.1038/nrc3130 21941284 PMC3307543

[B9] Bélanger C. Hould F. S. Lebel S. Biron S. Brochu G. Tchernof A. (2006). Omental and subcutaneous adipose tissue steroid levels in obese men. *Steroids* 71 674–682. 10.1016/j.steroids.2006.04.008 16762384

[B10] Ben Ali S. Kallel A. Sediri Y. Feki M. Slimane H. Jemaa R. (2009). Association of G-2548A LEP polymorphism with plasma leptin levels in Tunisian obese patients. *Clin. Biochem.* 42 584–588. 10.1016/j.clinbiochem.2008.11.001 19041299

[B11] Benton M. C. Johnstone A. Eccles D. Harmon B. Hayes M. T. Lea R. A. (2015). An analysis of DNA methylation in human adipose tissue reveals differential modification of obesity genes before and after gastric bypass and weight loss. *Genome. Biol.* 16:8 10.1186/s13059-014-0569-x PMC4301800 25651499

[B12] Bhaskaran K. Douglas I. Forbes H. dos-Santos-Silva I. Leon D. A. Smeeth L. (2014). Body-mass index and risk of 22 specific cancers: a population-based cohort study of 5.24 million UK adults. *Lancet* 384 755–765. 10.1016/S0140-6736(14)60892-8 25129328 PMC4151483

[B13] Bird A. (2002). DNA methylation patterns and epigenetic memory. *Genes. Dev.* 16 6–21. 10.1101/gad.947102 11782440

[B14] Björntorp P. (1995). Endocrine abnormalities in obesity. *Metabolism.* 44(9 Suppl. 3), 21–23. 10.1016/0026-0495(95)90315-1 7674912

[B15] Blüher M. (2009). Adipose tissue dysfunction in obesity. *Exp. Clin. Endocrinol. Diabetes* 117 241–250. 10.1055/s-0029-1192044 19358089

[B16] Bostrom M. A. Freedman B. I. Langefeld C. D. Liu L. Hicks P. J. Bowden D. W. (2009). Association of adiponectin gene polymorphisms with type 2 diabetes in an African American population enriched for nephropathy. *Diabetes Metab. Res. Rev.* 58 499–504. 10.2337/db08-0598 PMC2628626 19056609

[B17] Bouchard L. Tchernof A. Deshaies Y. Marceau S. Lescelleur O. Biron S. (2007). ZFP36:a promosing candidate gene for obesity-related metabolic complications identified by converging genomics. *Obes. Surg.* 17 372–382. 10.1007/s11695-007-9067-5 17546847

[B18] Bray F. Ren J. S. Masuyer E. Ferlay J. (2013). Estimates of global cancer prevalence for 27 sites in the adult population in 2008. *Int. J. Cancer* 132 1133–1145. 10.1002/ijc.27711 22752881

[B19] Bruemmer D. (2012). Targeting angiogenesis as treatment for obesity. *Arterioscler. Thromb. Vasc. Biol.* 32 161–162. 10.1161/ATVBAHA.111.241992 22258895

[B20] Bruun J. M. Lihn A. S. Pedersen S. B. Richelsen B. (2005). Monocyte chemoattractant Protein-1 release is higher in visceral than subcutaneous human adipose tissue (AT): implication of macrophages resident in the AT. *J. Clin. Endocrinol. Metab.* 90 2282–2289. 10.1210/jc.2004-1696 15671098

[B21] Cabia B. Andrade S. Carreira M. C. Casanueva F. F. Crujeiras A. B. (2016). A role for novel adipose tissue-secreted factors in obesiy-related carcinogenesis. *Obes. Rev.* 17 361–376. 10.1111/obr.12377 26914773

[B22] Cai C. Wang C. Ji W. Liu B. Kang Y. Hu Z. (2013). Knockdown of hepatic aquaglyceroporin-9 alleviates high fat diet-induced non-alcoholic fatty liver disease in rats. *Int. Immunopharmocol.* 15 550–556. 10.1016/j.intimp.2013.01.020 23415870

[B23] Calle E. E. Rodriguez C. Walker-Thurmond K. Thun M. J. (2003). Overweight, obesity, and mortality from cancer in a prospectively studied cohort of U.S. adults. *N. Engl. J. Med.* 348 1625–1638. 10.1056/NEJMoa021423 12711737

[B24] Campbell S. E. Febbraio M. A. (2002). Effect of the ovarian hormones on GLUT4 expression and contraction-stimulated glucose uptake. *Am. J. Physiol. Endocrinol. Metab.* 282 E1139–E1146. 10.1152/ajpendo.00184.2001 11934680

[B25] Catalán V. Gómez-Ambrosi J. Pastor C. Rotellar F. Silva C. Rodriguez A. (2008). Influence of morbid obesity and insulin resistance on gene expression levels of AQP7 in visceral adipose tissue and AQP9 in liver. *Obes. Surg.* 18 695–701. 10.1007/s11695-008-9453-7 18401671

[B26] Ceperuelo-Mallafré V. Miranda M. Chacón M. R. Vilarrasa N. Megia A. Gutiérrez C. (2007). Adipose tissue expression of the glycerol channel aquaporin-7 gene is altered in severe obesity but not in type 2 diabetes. *J. Clin. Endocrinol. Metab.* 92 3640–3645. 10.1210/jc.2007-0531 17566090

[B27] Chalasani N. Younossi Z. Lavine J. E. Diehl A. M. Brunt E. M. Cusi K. (2012). The diagnosis and management of non-alcoholic fatty liver disease: practice guideline by the American Gastroenterological Association, American Association for the Study of Liver Diseases, and American College of Gastroenterology. *Gastroenterology* 55 2005–2023. 10.1002/hep.25762 22488764

[B28] Chang Y. Jung H. S. Cho J. Zhang Y. Yun K. E. Lazo M. (2016). Metabolically healthy obesity and the development of nonalcoholic fatty liver disease. *Am. J. Gastroenterol.* 111 1133–1140. 10.1038/ajg.2016.178 27185080

[B29] Chatterjee T. K. Basford J. E. Yiew K. H. Stepp D. W. Hui D. Y. Weintraub N. L. (2014). Role of histone deacetylase 9 in regulating adipogenic differentiation and high fat-diet induced metabolic disease. *Adipocyte* 3 333–338. 10.4161/adip.28814 26317058 PMC4550687

[B30] Chatterjee T. K. Idelman G. Blanco V. Blomkalns A. L. Piegore M. G. Jr. Weintraub D. S. (2011). Histone deacetylase 9 is a negative reulator of adipogenic differentiation. *J. Biol. Chem.* 286 27836–27847. 10.1074/jbc.M111.262964 21680747 PMC3149373

[B31] Chen C. Chang Y. C. Liu C. L. Liu T. P. Chang K. J. Guo I. C. (2007). Leptin induces proliferation and anti-apoptosis in human hepatocarcinoma cells by up-regulating cyclin D1 and down-regulating Bax via a Janus kinase 2-linked pathway. *Endocr. Relat. Cancer* 14 513–529. 10.1677/ERC-06-0027 17639064

[B32] Chen M. J. Yeh Y. T. Lee K. T. Tsai C. J. Lee H. H. Wang S. N. (2012). The promoting effect of adiponectin in hepatocellular carcinoma. *J. Surg. Oncol.* 106 181–187. 10.1002/jso.23059 22287480

[B33] Chen X. Li C. Lü L. Mei Z. (2016). Expression and clinical significance of aquaglyceroporins in human hepatocellular carcinoma. *Mol. Med. Rep.* 13 5283–5289. 10.3892/mmr.2016.5184 27121567

[B34] Clore J. N. Glickman P. S. Helm S. T. Nestler J. E. Blackard W. G. (1989). Accelerated decline in hepatic glucose production during fasting in normal women compared with men. *Metabolism* 38 1103–1107. 10.1016/0026-0495(89)90047-4 2811679

[B35] Cowden Dahl K. D. Dahl R. Kruichak J. N. Hudson L. G. (2009). The epidermal growth factor receptor responsive miR-125a represses mesenchymal morphology in ovarian cancer cells. *Neoplasia* 11 1208–1215. 10.1593/neo.09942 19881956 PMC2767222

[B36] Dahlhoff M. Pfister S. Blutke A. Rozman J. Klingenspor M. Deutsch M. J. (2014). Peri-conceptional obesogenic exposure induces sex-specific programming of disease susceptibilities in adult mouse offspring. *Biochim. Biophys. Acta* 1842 304–317. 10.1016/j.bbadis.2013.11.021 24275555

[B37] Dahlman I. Sinha I. Gao H. Brodin D. Thorell A. Rydén M. (2015). The fat cell epigenetic signature in post-obese women is characterized by global hypomethylation and differential DNA methylation of adipogenesis genes. *Int. J. Obes. (Lond.)* 39 910–919. 10.1038/ijo.2015.31 25783037

[B38] Davis S. N. Shavers C. Costa F. (2000). Differential gender responses to hypoglycemia are due to alterations in CNS drive and not glycemic thresholds. *Am. J. Physiol. Endocrinol. Metab.* 279 E1054–E1063. 11052960 10.1152/ajpendo.2000.279.5.E1054

[B39] Deiuliis J. A. (2016). MicroRNAs as regulators of metabolic disease: pathophysiologic significance and emerging biomarkers and therapeutics. *Int. J. Obes.* 40 88–101. 10.1038/ijo.2015.170 PMC4722234 26311337

[B40] Derynck R. Zhang Y. Feng X. H. (1998). Smads: transcriptional activators of TGF-beta responses. *Cell* 95 737–740. 10.1016/S0092-8674(00)81696-7 9865691

[B41] Dick K. J. Nelson C. P. Tsaprouni L. Sandling J. K. Aïssi D. Wahl S. (2014). DNA methylation and body-mass index: a genome-wide analysis. *Lancet* 383 1990–1998. 10.1016/S0140-6736(13)62674-4 24630777

[B42] Dolley G. Bertrais S. Frochot V. Bebel J. F. Guerre-Millo M. Tores F. (2008). Promoter adiponectin polymorphisms and waist/hip ratio variation in a prospective French adults study. *Int. J. Obes. (Lond.)* 32 669–675. 10.1038/sj.ijo.0803773 18071343

[B43] Duan X. F. Tang P. Li Q. Yu Z. T. (2013). Obesity, adipokines and hepatocellular carcinoma. *Int. J. Cancer* 133 1776–1783. 10.1002/ijc.28105 23404222

[B44] El-Serag H. B. (2012). Epidemiology of viral hepatitis and hepatocellular carcinoma. *Gastroenterology* 142 1264–1273. 10.1053/j.gastro.2011 22537432 PMC3338949

[B45] ENCODE Project Consortium (2012). An integrated encyclopedia of DNA elements in the human genome. *Nature* 489 57–74. 10.1038/nature11247 22955616 PMC3439153

[B46] Esteller M. (2007). Cancer epigenomics: DNA methylomes and histone-modification maps. *Nat. Rev. Genet.* 8 286–298. 10.1038/nrg2005 17339880

[B47] Fairfax B. P. Makino S. Radhakrishnan J. Plant K. Leslie S. Dilthey A. (2012). Genetics of gene expression in primary immune cells identifies cell type-specific master regulators and roles of HLA alleles. *Nat. Genet.* 44 502–510. 10.1038/ng.2205 22446964 PMC3437404

[B48] Farazi P. A. DePinho R. A. (2006). Hepatocellular carcinoma pathogenesis: from genes to environment. *Nat. Rev. Cancer* 6 674–687. 10.1038/nrc1934 16929323

[B49] Ferlay J. Soerjomataram I. Dikshit R. Eser S. Mathers C. Rebelo M. (2015). Cancer incidence and mortality worldwide: sources, methods and major patterns in GLOBOCAN 2012. *Int. J. Cancer* 136 E359–E386. 10.1002/ijc.29210 25220842

[B50] Ferrente A. W. Jr. (2013). The immune cells in adipose tissue. *Diabetes Obes. Metab.* 15 34–38. 10.1111/dom.12154 24003919 PMC3777665

[B51] Finucane M. M. Stevens G. A. Cowan M. J. Danaei G. Paciorek C. J. Singh G. M. (2011). National, regional and global trends in body-mass index since 1980: systematic analysis of health examination surveys and epidemiological studies with 960 country-years and 9.1 million participants. *Lancet* 377 557–567. 10.1016/S0140-6736(10)62037-5 21295846 PMC4472365

[B52] Fraga M. F. Ballestar E. Paz M. F. Ropero S. Setien F. Ballestar M. L. (2005). Epigenetic differences arise during the lifetime of monozygotic twins. *Proc. Natl. Acad. Sci. U.S.A.* 102 10604–10609. 10.1073/pnas.0500398102 16009939 PMC1174919

[B53] Franckhauser S. Munoz S. Elias I. Ferre T. Bosch F. (2006). Adipose overexpression of phosphoenolpyruvate carboxykinase leads to high susceptibility to diet-induced insulin resistance and obesity. *Diabetes Metab. Res. Rev.* 55 273–280. 10.2337/diabetes.55.02.06.db05-0482 16443757

[B54] Freedland E. S. (2004). Roles of critical visceral adipose tissue threshold in metabolic syndrome: implications for controlling dietary carbohydrates: a review. *Nutr. Metab.* 1:12 10.1186/1743-7075-1-12 PMC535537 15530168

[B55] Frühbeck G. (2005). Obesity: aquaporin enters the picture. *Nature* 438 436–437. 10.1038/438436b 16306977

[B56] Frühbeck G. Gómez-Ambrosi J. Salvador J. (2001). Leptin-induced lipolysis opposes the tonic inhibition of endogenous adenosine in white adipocytes. *FASEB J.* 15 333–340. 10.1096/fj.00-0249com 11156949

[B57] Frühbeck G. Méndez-Giménez L. Fernández-Formoso J. A. Fernández S. Rodríguez A. (2014). Regulation of adipocyte lipolysis. *Nutr. Res. Rev.* 27 63–93. 10.1017/S095442241400002X 24872083

[B58] Fujiwara N. Nakagawa H. Kudo Y. Tateishi R. Taguri M. Watadani T. (2015). Sarcopenia, intramuscular fat deposition, and viscaeral adiposity independently predict the outcomes of hepatocellular carcinoma. *J. Hepatol.* 63 131–140. 10.1016/j.jhep.2015.02.031 25724366

[B59] Garaulet M. Pérez-Llamas F. Fuente T. Zamora S. Tebar F. J. (2000). Anthropometric, computed tomography and fat cell data in an obese population: relationship with insulin, leptin, tumor necrosis factor-alpha, sex hormone-binding globulin and sex hormones. *Eur. J. Endocrinol.* 143 657–666. 10.1530/eje.0.1430657 11078990

[B60] GBD 2013 Mortality and Causes of Death Collaborators (2015) Global, regional, and national age-sex specific all-cause and cause-specific mortality for 240 causes of death, 1990-2013: a systematic analysis for the Global Burden of Disease Study 2013. *Lancet* 385 117–171. 10.1016/S0140-6736(14)61682-2 25530442 PMC4340604

[B61] Ge Z. Luo S. Lin F. Liang Q. Huang L. Wei Y. (2014). DNA methylation in oocytes and live of female mice and their offspring: effects of high-fat-diet-induced obesity. *Environ. Health Perspect.* 122 159–164. 10.1289/ehp.1307047 24316659 PMC3915265

[B62] Gregor M. F. Hotamisligil G. S. (2011). Inflammatory mechanisms in obesity. *Annu. Rev. Immunol.* 29 415–445. 10.1146/annurev-immunol-031210-101322 21219177

[B63] Guerrero R. Vega G. L. Grundy S. M. Browning J. D. (2009). Ethnic differences in hepatic steatosis: an insulin resistance paradox? *Hepatology* 49 791–801. 10.1002/hep.22726 19105205 PMC2675577

[B64] Guo S. Lu J. Schlanger R. Zhang H. Wang J. Y. Fox M. C. (2010). MicroRNA miR-125a controls hematopoietic stem cell number. *Proc. Natl. Acad. Sci. U.S.A.* 107 14229–14234. 10.1073/pnas.0913574107 20616003 PMC2922532

[B65] Hardo O. T. Perugini R. A. Nicoloro S. M. Gallagher-Doval K. Purl V. Straubhaar J. (2011). Body mass index-independent inflammation in omental adipose tissue associated with insulin resistance in morbid obesity. *Surg. Obes. Relat. Dis.* 7 60–67. 10.1016/j.soard.2010.05.013 20678967 PMC2980798

[B66] Hatanaka M. Shimba S. Sakaue M. Kondo Y. Kagechika H. Kokame K. (2009). Hypoxia-inducible factor 3α functions as an accelerator of 3T3-L1 adipose differentiation. *Biol. Pharm. Bull.* 32 1166–1172. 10.1248/bpb.32.1166 19571379

[B67] Hedrington M. S. Davis S. N. (2015). Sexual dimorphism in glucose and lipid metabolism during fasting, hypoglycemia, and exercise. *Front. Endocrinol. (Lausanne)* 6:61 10.3389/fendo.2015.00061 PMC4410598 25964778

[B68] Heidbreder M. Qadri F. Jöhren O. Dendorfer A. Depping R. Fröhlich F. (2007). Non-hypoxic induction of HIF-3α by 2-deoxy-D-glycose and insulin. *Biochem. Biophys. Res. Commun.* 352 437–443. 10.1016/j.bbrc.2006.11.027 17125738

[B69] Herrera B. M. Lockstone H. E. Taylor J. M. Ria M. Barrett A. Collins S. (2010). Global microRNA expression profiles in insulin target tissues in a spontaneous rat model of type 2 diabetes. *Diabetologia* 53 1099–1109. 10.1007/s00125-010-1667-2 20198361 PMC2860560

[B70] Herrera B. M. Lockstone H. E. Taylor J. M. Wills Q. F. Kaisaki P. J. Barrett A. (2009). MicroRNA-125a is over-expressed in insulin target tissues in a spontaneous rat model of Type 2 diabetes. *BMC Med. Genomics.* 2:54 10.1186/1755-8794-2-54 PMC2754496 19689793

[B71] Hibuse T. Maeda N. Funahashi T. Yamamoto K. Nagasawa A. Mizunoya W. (2005). Aquaporin 7 deficiency is associated with development of obesity through activation of adipose glycerol kinase. *Proc. Natl. Acad. Sci. U.S.A.* 102 10993–10998. 10.1073/pnas.0503291102 16009937 PMC1182435

[B72] Housa D. Housová J. Vernerová Z. Haluzík M. (2006). Adipocytokines and cancer. *Physiol. Res.* 55 233–244. 16238454 10.33549/physiolres.930848

[B73] Huss J. M. Torra I. P. Staels B. Giguere V. Kelly D. P. (2004). Estrogen-related receptor alpha directs peroxisome proliferator-activated receptor alpha signaling in the transcriptional control of energy metabolism in cardiac and skeletal muscle. *Mol. Cell. Biol.* 24 9079–9091. 10.1128/MCB.24.20.9079-9091.2004 15456881 PMC517878

[B74] Ibrahim M. M. (2010). Subcutaneous and visceral adipose tissue: structural and functional differences. *Obes. Rev.* 11 11–18. 10.1111/j.1467-789X.2009.00623.x 19656312

[B75] Ikejima K. Honda H. Yoshikawa M. Hirose M. Kitamura T. Takei Y. (2001). Leptin augments inflammatory and profibrogenic responses in the murine liver induced by hepatotoxic chemicals. *Hepatology* 34 288–297. 10.1053/jhep.2001.26518 11481614

[B76] Isobe T. Saitoh S. Takagi S. Takeuchi H. Chiba Y. Katoh N. (2005). Influence of gender, age and renal function on plasma adiponectin level: the Tanno and Sobetsu study. *Eur. J. Endocrinol.* 153 91–98. 10.1530/eje.1.01930 15994750

[B77] Iwafuchi-Doi M. Donahue G. Kakumanu A. Watts J. A. Mahony S. Franklin Pugh B. (2016). The pioneer transcription factor FoxA maintains an accessible nucleosome configuration at enhancers for tissue-specific gene activation. *Mol. Cell.* 62 79–91. 10.1016/j.molcel.2016.03.001 27058788 PMC4826471

[B78] Jablonski E. M. Mattocks M. A. Sokolov E. Koniaris L. G. Hughes F. M. Jr. (2007). Decreased aquaporin expression leads to increased resistance to apoptosis in hepatocellular carcinoma. *Cancer Lett.* 250 36–46. 10.1016/j.canlet.2006.09.013 17084522 PMC1934939

[B79] Ji H. L. Song C. C. Li Y. F. He J. J. Zheng X. L. Yang G. S. (2014). miR-125a inhibits porcine preadipocytes differentiation by targeting ERRα. *Mol. Cell. Biochem.* 395 155–165. 10.1007/s11010-014-2121-4 24952481

[B80] Jiang L. Huang Q. Zhang S. Zhang Q. Chang J. Qiu X. (2010). Hsa-miR-125a-3p and hsa-miR-125a-5p are downregulated in non-small cell lung cancer and have inverse effects on invasion and migration of lung cancer cells. *BMC Cancer.* 10:318 10.1186/1471-2407-10-318 PMC2903529 20569443

[B81] Klein S. (2004). The case of visceral fat: argument for the defense. *J. Clin. Invest.* 113 1530–1532. 10.1172/JCI200422028 15173878 PMC419497

[B82] Klein S. Flanagan L. (2016). Sex differences in immune responses. *Nat. Rev. Immunol.* 10.1038/nri.2016.90 [Epub ahead of print]. 27546235

[B83] Klimcakova E. Roussel B. Kovacova Z. Kovacikova M. Siklova-Vitkova M. Combes M. (2011). Macrophage gene expression is related to obesity and the metabolic syndrome in human subcutaneous fat as well as in visceral fat. *Diabetologia* 54 876–887. 10.1007/s00125-010-2014-3 21267541

[B84] Kraus M. Greither T. Wenzel C. Bräuer-Hartmann D. Wabitsch M. Behre H. M. (2015). Inhibition of adipogenic differentiation of human SGBS preadipocytes by androgen-regulated microRNA miR-375. *Mol. Cell. Endocrinol.* 414 177–185. 10.1016/j.mce.2015.07.026 26219823

[B85] Krek A. Grün D. Poy M. N. Wolf R. Rosenberg L. Epstein E. J. (2005). Combinatorial microRNA target predictions. *Nat. Genet.* 37 495–500. 10.1038/ng1536 15806104

[B86] Kunnari A. Santaniemi M. Jokela M. Karjalainen A. H. Heikknen J. Ukkola O. (2008). Estrogen replacement therapy decreases plasma adiponectin but not resistin in postmenopausal women. *Metabolism* 57 1509–1515. 10.1016/j.metabol.2008.06.004 18940387

[B87] Kuriyama H. Shimomura I. Kishida K. Kondo H. Furuyama N. Nishizawa H. (2002). Coordinated regulation of fat-specific and liver-specific glycerol channels, aquaporin adipose and aquaporin 9. *Diabetes* 51 2915–2921. 12351427 10.2337/diabetes.51.10.2915

[B88] Kuroda M. Tominaga A. Nakagawa K. Nishiguchi M. Sebe M. Miyatake Y. (2016). DNA methylation suppresses leptin gene in 3T3-L1 adipocytes. *PLoS ONE* 11:e0160532 10.1371/journal.pone.0160532 PMC4975473 27494408

[B89] Laforenza U. Scaffino M. F. Gastaldi G. (2013). Aquaporin-10 represents an alternative pathway for glycerol efflux from human adipocytes. *PLoS ONE* 8:e54474 10.1371/journal.pone.0054474 PMC3558521 23382902

[B90] Lanfranco F. Zitzmann M. Simoni M. Nieschlag E. (2004). Serum adiponectin levels in hypogonadal males: influence of testosterone replacement therapy. *Clin. Endocrinol. (Oxf.)* 60 500–507. 10.1111/j.1365-2265.2004.02007.x 15049966

[B91] Laughlin G. A. Barrett-Connor E. May S. (2006). Sex-specific association of the androgen to oestrogen ratio with adipocytokine levels in older adults: the rancho bernardo study. *Clin. Endocrinol. (Oxf.)* 65 506–513. 10.1111/j.1365-2265.2006.02624.x 16984244

[B92] Lê K. Mahurkar S. Alderete T. L. Hasson R. E. Adam T. C. Kim J. S. (2011). Subcutaneous adipose tissue macrophage infiltration is associated with hepatic and visceral fat deposition, hyperinsulinemia, and stimulation of NF-κB stress pathway. *Diabetes Metab. Res. Rev.* 60 2802–2809. 10.2337/db10-1263 PMC3198061 22025778

[B93] Li C. Zhang W. Liu M. Qiu L. Chen X. Lv L. (2016). Aquaporin 9 inhibits hepatocellular carcinoma through up-regulating FOXO1 expression. *Oncotarget* 7 44161–44170. 10.18632/oncotarget.10143 PMC5190086 27329843

[B94] Li Z. Tuteja G. Schug J. Kaestner K. H. (2012). Foxa1 and Foxa2 are essential for sexual dimorphism in liver cancer. *Cell* 148 72–83. 10.1016/j.cell.2011.11.026 22265403 PMC3266536

[B95] Licinio J. Negrão A. B. Mantzoros C. Kaklamani V. Wong M. L. Bongiorno P. B. (1998). Sex differences in circulating human leptin pulse amplitude: clinical implications. *J. Clin. Endocrinol. Metab.* 83 4140–4147. 10.1210/jcem.83.11.5291 9814504

[B96] Ling H. Y. Wen G. B. Feng S. D. Tuo Q. H. Ou H. S. Yao C. H. (2011). MicroRNA-375 promotes 3T3-L1 adipocyte differentiation through modulation of extracellular signal-regulated kinase signaling. *Clin. Exp. Pharmacol. Physiol.* 38 239–246. 10.1111/j.1440-1681.2011.05493.x 21291493 PMC3086632

[B97] Lira F. S. Rosa J. C. Pimentel G. D. Seelaender M. Damaso A. R. Oyama L. M. (2012). Both adiponectin and interleukin-10 inhibit LPS-induced activation of the NF-κB pathway in 3T3-L1 adipocytes. *Cytokine* 57 98–106. 10.1016/j.cyto.2011.10.001 22047972

[B98] Liu P. Shi H. Huang C. Shu H. Liu R. Yang Y. (2014). Association of LEP A19G polymorphism with cancer risk: a systematic review and pooled analysis. *Tumor. Biol.* 35 8133–8141. 10.1007/s13277-014-2088-5 24845032

[B99] Livingstone C. Collison M. (2002). Sex steroids and insulin resistance. *Clin. Sci. (Lond.)* 102 151–166. 10.1042/cs1020151 11834135

[B100] Lumeng C. N. Bodzin J. L. Saltiel A. R. (2007). Obesity induces in phenotypic switch in adipose tissue macrophage polarization. *J. Clin. Invest.* 117 175–184. 10.1172/JCI29881 17200717 PMC1716210

[B101] Ma C. Kesarwala A. H. Eggert T. Medina-Echeverz J. Kleiner D. E. Jin P. (2016). NAFLD causes selective CD4+ T lymphocytes loss and promotes hepatocarcinogenesis. *Nature* 531 253–257. 10.1038/nature16969 26934227 PMC4786464

[B102] Ma W. L. Hsu C. L. Yeh C. C. Wu M. H. Huang C. K. Jeng L. B. (2012). Hepatic androgen receptor suppresses hepatocellular carcinoma metastasis through modulation of cell migration and anoikis. *Hepatology* 56 176–185. 10.1002/hep.25644 22318717 PMC3673306

[B103] Ma Y. Liu D. (2013). Hydrodynamic deliver of adiponectin and adiponectin receptor 2 gene blocks high-fat diet-induced obesity and insulin resistance. *Gene. Ther.* 20 846–852. 10.1038/gt.2013.8 23425917 PMC3740076

[B104] Machinal F. Dieudonne M. N. Leneveu M. Pecquery R. Giudicelli Y. (1999). In vivo and in vitro ob gene expression and leptin secretion in rat adipocytes: evidence for a regional specific regulation by sex steroid hormones. *Endocrinology* 140 1567–1574. 10.1210/endo.140.4.6617 10098489

[B105] Mackevics V. Heid I. M. Wagner S. A. Cip P. Doppelmayr H. Lejnieks A. (2006). The adiponectin gene is associated with adiponectin levels but not with characteristics of the insulin resistance syndrome in healthy Caucasians. *Eur. J. Hum. Genet.* 14 349–356. 10.1038/sj.ejhg.5201552 16418740

[B106] Madeira A. Fernández-Veledo S. Camps M. Zorzano A. Moura T. F. Ceperuelo-Mallafré V. (2014). Human aquaporin-11 is a water and glycerol channel and localizes in the vicinity of lipid droplets in human adipocytes. *Obesity* 22 2010–2017. 10.1002/oby.20792 24845055

[B107] Marcello M. A. Calixto A. R. de Almeida J. F. Martins M. B. Cunha L. L. Cavalari C. A. (2015). Polymorphism in LEP and LEPR may modify leptin levels and represent risk factors for thyroid cancer. *Int. J. Endocrinol.* 2015:173218 10.1155/2015/173218 PMC4355553 25810718

[B108] Marrades M. P. Milagro F. I. Martínez J. A. Moreno-Aliaga M. J. (2006). Differential expression of aquaporin 7 in adipose tissue of lean and obese high fat consumers. *Biochem. Biophys. Res. Commun.* 339 785–789. 10.1016/j.bbrc.2005.11.080 16325777

[B109] Martínez J. A. Milagro F. I. Claycombe K. J. Schalinske K. L. (2014). Epigenetics in adipose tissue, obesity, weight loss, and diabetes. *Adv. Nutr.* 5 71–81. 10.3945/an.113.004705 24425725 PMC3884103

[B110] Matsumura Y. Nakaki R. Inagaki T. Yoshida A. Kano Y. Kimura H. (2015). H3K4/H3K9me3 bivalent chromatin domains targeted by lineage-specific DNA methylation pauses adipocyte differentiation. *Mol. Cell* 60 584–596. 10.1016/j.molcel.2015.10.025 26590716

[B111] McKeigue P. M. Shah B. Marmot M. G. (1991). Relation of central obesity and insulin resistance with high diabetes prevalence and cardiovascular risk in South Asians. *Lancet* 337 382–386. 10.1016/0140-6736(91)91164-P 1671422

[B112] Melzner I. Scott V. Dorsch K. Fischer P. Wabitsch M. Brüderlein S. (2002). Leptin gene expression in human preadipocytes is switched on by maturation-induced demethylation of distinct CpGs in its proximal promoter. *J. Biol. Chem.* 277 45420–45427. 10.1074/jbc.M208511200 12213831

[B113] Mendell J. T. (2005). MicroRNAs: critical regulators of development, cellular physiology and malignancy. *Cell. Cycle* 4 1179–1184. 10.4161/cc.4.9.2032 16096373

[B114] Michaud A. Drolet R. Noël S. Paris G. Tchernof A. (2012). Visceral fat accumulation is an indicator of adipose tissue macrophage infiltration in women. *Metabolism* 61 689–698. 10.1016/j.metabol.2011.10.004 22154325

[B115] Miranda M. Escoté X. Ceperuelo-Mallafré V. Alcaide M. J. Simón I. Vilarrasa N. (2010). Paired subcutaneous and visceral adipose tissue aquaporin-7 expression in human obesity and type 2 diabetes: differences and similarities between depots. *J. Clin. Endocrinol. Metab.* 95 3470–3479. 10.1210/jc.2009-2655 20463097

[B116] Naugler W. E. Sakurai T. Kim S. Maeda S. Kim K. Elsharkawy A. M. (2007). Gender disparity in liver cancer due to sex differences in MyD88-dependent IL-6 production. *Science* 317 121–124. 10.1126/science.1140485 17615358

[B117] NCD Risk Factor Collaboration [NCD-RisC] (2016). Worldwide treands in diabetes since 1980: a pooled analysis of 751 population0based studies with 4. 4 million. (participants). *Lancet* 387 1513–1530. 10.1016/S0140-6736(16)00618-8 27061677 PMC5081106

[B118] Nishizawa H. Shimomura I. Kishida K. Maeda N. Kuriyama H. Nagaretani H. (2002). Androgens decrease plasma adipnectin, an insulin-sensitizing adipocyte-derived protein. *Diabetes Metab. Res. Rev.* 51 2734–2741. 10.2337/diabetes.51.9.2734 12196466

[B119] Nohara K. Laque A. Allard C. Münzberg H. Mauvais-Jarvis F. (2014). Central mechanisms of adiposity in adult female mice with androgen excess. *Obesity (Silver Spring)* 22 1477–1484. 10.1002/oby.20719 24639082 PMC4037375

[B120] Ohki T. Tateishi R. Shiina S. Goto E. Sato T. Nakagawa H. (2009). Visceral fat accumulation is an independent risk factor for hepatocellular carcinoma recurrence after curative treatment in patients with suspected NASH. *Gut* 58 839–844. 10.1136/gut.2008.164053 19174415

[B121] Okwan-Duodu D. Umpierrez G. E. Brawley O. W. Diaz R. (2013). Obesity-driven inflammation and cancer risk: role of myeloid derived suppressor cells and alternately activated macrophages. *Am. J. Cancer. Res.* 3 21–33. 23359288 PMC3555202

[B122] Ortega F. J. Moreno-Navarrete J. M. Pardo G. Sabater M. Hummel M. Ferrer A. (2010). MiRNA expression profile of human subcutaneous adipose and during adipocyte differentiation. *PLoS ONE* 5:e9022 10.1371/journal.pone.0009022 PMC2814866 20126310

[B123] Padma S. Smeltz A. M. Banks P. M. Iannitti D. A. McKillop I. H. (2009). Altered aquaporin 9 expression and localization in human hepatocellular carcinoma. *HPB (Oxford)* 11 66–74. 10.1111/j.1477-2574.2008.00014.x 19590626 PMC2697857

[B124] Page S. T. Herbst K. L. Amory J. K. Coviello A. D. Anawalt B. D. Bremner W. J. (2005). Testosterone administration suppresses adiponectin levels in men. *J. Androl.* 26 85–92. 15611571

[B125] Panguluri S. K. Tur J. Chapalamadugu K. C. Katnik C. Cuevas J. Tipparaju S. M. (2013). MicroRNA-301a mediated regulation of Kv4.2 in diabetes: identification of key modulators. *PLoS ONE* 8:e60545 10.1371/journal.pone.0060545 PMC3616003 23573265

[B126] Park E. J. Lee J. H. Yu G. Y. He G. Ali S. R. Holzer R. G. (2010). Dietary and genetic obesity promote liver inflammation and tumorigenesis by IL-6 and TNF expression. *Cell* 140 197–208. 10.1016/j.cell.2009.12.052 20141834 PMC2836922

[B127] Parkin D. M. Bray J. Ferlay P. Pisani P. (2006). Global cancer statistics. *CA Cancer J. Clin.* 55 74–108. 10.3322/canjclin.55.2.74 15761078

[B128] Paschos P. Paletas K. (2009). Non alcoholic fatty liver disease and metabolic syndrome. *Hippokratia* 13 9–19. 19240815 PMC2633261

[B129] Peng R. Zhao G. Li J. Zhang Y. Shen X. Wang J. (2016). Auphen and dibutyryl cAMP suppress growth of hepatocellular carcinoma by regulating expression of aquaporins 3 and 9 in vivo. *World J. Gastroenterol.* 22 3341–3354. 10.3748/wjg.v22.i12.3341 27022216 PMC4806192

[B130] Pérez-Pérez R. Ortega-Delgado F. J. García-Santos E. López J. A. Camafeita E. Ricart W. (2009). Differential proteomics of omental and subcutaneous adipose tissue reflects their unalike biochemical and metabolic properties. *J. Proteome. Res.* 8 1682–1693. 10.1021/pr800942k 19714809

[B131] Peters K. E. Beilby J. Cadby G. Warrington N. M. Bruce D. G. Davis W. A. (2013). A comprehensive investigation of variants in genes encoding adiponectin (ADIPOQ) and its receptors (ADIPOR1/R2), and their association with serum adiponectin, type 2 diabetes, insulin resistance and the metabolic syndrome. *BMC Med. Genet.* 14:15 10.1186/1471-2350-14-15 PMC3598639 23351195

[B132] Petrangeli E. Coroniti G. Brini A. T. de Girolamo L. Stanco D. Niada S. (2016). Hypoxia promotes the inflammatory response and stemness features in visceral fat stem cells from obese subjects. *J. Cell. Physiol.* 231 668–679. 10.1002/jcp.25113 26224080

[B133] Qi W. Gao S. Wang Z. (2008). Transcriptional regulation of the TGrF-beta1 promoter by androgen receptor. *Biochem. J.* 416 453–462. 10.1042/BJ20080651 18651839

[B134] Rama-Esendagli D. Esendagli G. Yilmaz G. Guc D. (2014). Spheroid formation and invasion capacity are differentially influenced by co-cultures of fibroblast and macrophage cells in breast cancer. *Mol. Biol. Rep.* 41 2885–2892. 10.1007/s11033-014-3144-3 24469725

[B135] Rankinen T. Zyberi A. Chagnon Y. C. Weisnagel S. J. Argyropoulos G. Walts B. (2006). The human obesity gene map: the 2005 update. *Obesity. (Silver Spring)* 14 529–644. 10.1038/oby.2006.71 16741264

[B136] Rao X. Di Leva G. Li M. Fang F. Devlin C. Hartman-Frey C. (2011). MicroRNA-221/222 confers breast cancer fulvestrant resistance by regulating multiple signaling pathways. *Oncogene* 30 1082–1097. 10.1038/onc.2010.487 21057537 PMC3342929

[B137] Reshef L. Olswang Y. Cassuto H. Blum B. Croniger C. M. Kalhan S. C. (2003). Glyceroneogenesis and the triglyceride/fatty acid cycle. *J. Biol. Chem.* 278 30413–30416. 10.1074/jbc.R300017200 12788931

[B138] Retnakaran R. Qi Y. Connelly P. W. Sermer M. Hanley A. J. Zinman B. (2010). Low adiponectin concentration during pregnancy predicts postpartum insulin resistance, β cell dysfunction and fasting glycaemia. *Diabetologia* 53 268–276. 10.1007/s00125-009-1600-8 19937225 PMC2878328

[B139] Ribatti D. Ranieri G. Annese T. Nico B. (2014). Aquaporins in cancer. *Biochim. Biophys. Acta* 1840 1550–1553. 10.1016/j.bbagen.2013.09.025 24064112

[B140] Riestra P. Gebreab S. Y. Xu R. Khan R. J. Bidulescu A. Correa A. (2015). Gender-specific associations between ADIPOQ gene polymorphisms and adiponectin levels and obesity in the Jackson Heart Study cohort. *BMC Med. Genet.* 16:65 10.1186/s12881-015-0214-x PMC4593213 26290432

[B141] Robciuc M. R. Naukkarinen J. Ortega-Alonso A. Tyynismaa H. Raivio T. Rissanen A. (2011). Serum angiopoietin-like 4 protein levels and expression in adipose tissue are inversely correlated with obesity in monozygotic twins. *J. Lipid. Res.* 52 1575–1582. 10.1194/jlr.P015867 21596930 PMC3137024

[B142] Rodríguez A. Catalán V. Gómez-Ambrosi J. Frühbeck G. (2006). Role of aquaporin-7 in the pathophysiology control of fat accumulation in mice. *FEBS Lett.* 580 4771–4776. 10.1016/j.febslet.2006.07.080 16919625

[B143] Rodríguez A. Catalán V. Gómez-Ambrosi J. Frühbeck G. (2011a). Aquaglyceroporins serve as metabolic gateways in adiposity and insulin resistance control. *Cell Cycle* 10 1548–1556. 10.4161/cc.10.10.15672 21502813 PMC3127157

[B144] Rodríguez A. Catalán V. Gómez-Ambrosi J. García-Navarro S. Rotellar F. Valentí V. (2011b). Insulin- and leptin-mediated control of aquaglyceroporins in human adipocytes and hepatocytes is mediated via the PI3K/Akt/mTOR signaling cascade. *J. Clin. Endocrinol. Metab.* 96 E586–E597. 10.1210/jc.2010-1408 21289260

[B145] Rodríguez A. Marinelli R. A. Tesse A. Frühbeck G. Calamita G. (2015a). Sexual dimorphism of adipose and hepatic aquaglyceroporins in health and metabolic disorders. *Front. Endocrinol.* 6:171 10.3389/fendo.2015.00171 PMC4633488 26594198

[B146] Rodríguez A. Moreno N. R. Balaguer I. Méndez-Giménez L. Becerril S. Catalán V. (2015b). Leptin administration restores the altered adipose and hepatic expression of aquaglyceroporins improving the non-alcoholic fatty liver of ob/ob mice. *Sci. Rep.* 5:12067 10.1038/srep12067 PMC4498231 26159457

[B147] Rojek A. M. Skowronski M. T. Füchtbauer E. M. Füchtbauer A. C. Fenton R. A. Agre Frøkiær J. (2007). Defective glycerol metabolism in aquaporin 9 (AQP9) knockout mice. *Proc. Natl. Acad. Soc. U.S.A.* 104 3609–3614. 10.1073/pnas.0610894104 PMC1805577 17360690

[B148] Rönn T. Volkov P. Davegärdh C. Dayeh T. Hall E. Olsson A. H. (2013). A six month exercise intervention influences the genome-wide DNA methylation pattern in human adipose tissue. *PLoS Genet.* 9:e1003572 10.1371/journal.pgen.1003572 PMC3694844 23825961

[B149] Rosen E. D. MacDougald O. A. (2006). Adipocyte differentiation from the inside out. *Nat. Rev. Mol. Cell. Biol.* 7 885–896. 10.1038/nrm2066 17139329

[B150] Sadik N. A. Ahmed A. Ahmed S. (2012). The significance of serum levels of adiponectin, leptin, and hyaluronic acid in hepatocellular carcinoma of cirrhotic and noncirrhotic patients. *Hum. Exp. Toxicol.* 31 311–321. 10.1177/0960327111431091 22249387

[B151] Sandovici I. Smith N. H. Nitert M. D. Ackers-Johnson M. Uribe-Lewis S. Ito Y. (2011). Maternal diet and agin alter the epigenetic control of a promoter-enhancer interaction at the Hnf4a gene in rat pancreatic islets. *Proc. Natl. Acad. Sci. U.S.A.* 108 5349–5354. 10.1073/pnas.1019007108 PMC3069181 21385945

[B152] Schlesinger S. Aleksandrova K. Pischon T. Fedirko V. Jenab M. Trepo E. (2013). Abdominal obesity, weight gain during adulthood and risk of liver and biliary tract cancer in a European cohort. *Int. J. Cancer* 132 645–657. 10.1002/ijc.27645 22618881

[B153] Schmidt S. L. Bessesen D. H. Stotz S. Peelor F. F. III Miller B. F. Horton T. J. (2014). Adrenergic control of lipolysis in women compared with men. *J. Appl. Physiol. (1985)* 117 1008–1019. 10.1152/japplphysiol.00003.2014 25190743 PMC4217046

[B154] Setiawan V. W. Lim U. Lipworth L. Lu S. C. Shepherd J. Ernst T. (2016). Sex and ethnic differences in the association of obesity with risk of hepatocellular carcinoma. *Clin. Gastroenterol. Hepatol.* 14 309–316. 10.1016/j.cgh.2015.09.015 26404865 PMC4718778

[B155] Shahid A. Rana S. Mahmood S. Saeed S. (2015). Role of leptin G-2548A polymorphism in age- and gender-specific development of obesity. *J. Biosci.* 40 521–530. 10.1007/s12038-015-9536-2 26333398

[B156] Sharma D. Wang J. Fu P. P. Sharma S. Nagalingam A. Mells J. (2010). Adiponectin antagonizes the oncogenic actions of leptin in hepatocellular carcinogenesis. *Hepatology* 52 1713–1722. 10.1002/hep.23892 20941777 PMC2967627

[B157] Shi Z. Zhao C. Guo X. Ding H. Cui Y. Shen R. (2014). Differential expression of microRNAs inomental adipose tissue from gestational diabetes mellitus subjects reveals miR-222 as a regulator of ERα expression in estrogen-induced insulin resistance. *Endocrinology* 155 1982–1990. 10.1210/en.2013-2046 24601884

[B158] Sjöholm K. Palming J. Olofsson L. E. Gummesson A. Svensson P. A. Lystig T. C. (2005). A microarray search for genes predominantly expressed in human omental adipocytes: adipose tissue as a major production site of serum amyloid A. *J. Clin. Endocrinol. Metab.* 90 2233–2239. 10.1210/jc.2004-1830 15623807

[B159] Slattery M. L. Wolff R. K. Herrick J. Caan B. J. Potter J. D. (2008). Leptin and leptin receptor genotypes and colon cancer: gene-gene and gene-lifestyle interactions. *Int. J. Cancer* 122 1611–1617. 10.1002/ijc.23135 18059035 PMC2430084

[B160] Spoto B. Di Betta E. Mattace-Raso F. Sijbbrands R. Vilardi A. Parlongo R. M. (2014). Pro- and anti-inflammatory cytokine gene expression in subcutaneous and visceral fat in severe obesity. *Nutr. Metab. Cardiovasc. Dis.* 24 1137–1143. 10.1016/j.numecd.2014.04.017 24984824

[B161] Sun T. Fu M. Bookout A. L. Kliewer S. A. Mangelsdorf D. J. (2009). MicroRNA let-7 regulates 3T3-L1 adipogenesis. *Mol. Endocrinol.* 23 925–931. 10.1210/me.2008-0298 19324969 PMC2691679

[B162] Sung Y. J. Pérusse L. Sarzynski M. A. Fornage M. Sidney S. Sternfeld B. (2016). Genome-wide association studies suggest sex-specific loci associated with abdominal and visceral fat. *Int. J. Obes.* 40 662–674. 10.1038/ijo.2015.217 PMC4821694 26480920

[B163] Tian Y. Wong V. W. Chan H. L. Cheng A. S. (2013). Epigenetic regulation of hepatocellular carcinoma in non-alcoholic fatty liver disease. *Sem. Cancer. Biol.* 23(6 Pt B), 471–482. 10.1016/j.semcancer.2013.08.010 24018165

[B164] Tian Y. Xie X. U. Lin Y. Tan G. Zhong W. U. (2015). Androgen receptor in hepatocarcinogenesis: recent developments and perspectives. *Oncol. Lett.* 9 1983–1988. 10.3892/ol.2015.3025 26136999 PMC4467293

[B165] Tiniakos D. G. Vos M. B. Brunt E. M. (2010). Nonalcoholic fatty liver disease: pathology and pathogenesis. *Annu. Rev. Pathol.* 5 145–171. 10.1146/annurev-pathol-121808-102132 20078219

[B166] Tobi E. W. Lumey L. H. Talens R. P. Kremer D. Putter H. Stein A. D. (2009). DNA methylation differences after exposure to prenatal famine are common and timing and sex-specific. *Hum. Mol. Genet.* 18 4046–4053. 10.1093/hmg/ddp353 19656776 PMC2758137

[B167] Trayhurn P. Wang B. Wood I. S. (2008). Hypoxia in adipose tissue: a basis for the dysregulation of tissue function in obesity. *Br. J. Nutr.* 100 227–235. 10.1017/S0007114508971282 18397542

[B168] Trayhurn P. Wood I. S. (2004). Adipokines: inflammation and pleiotropic role of white adipose tissue. *Br. J. Nutr.* 92 347–355. 10.1079/BJN20041213 15469638

[B169] Tsai E. C. Matsumoto A. M. Fujimoto W. Y. Boyko E. J. (2004). Association of bioavailable, free, and total testosterone with insulin resistance: influence of sex hormone-binding globulin and body fat. *Diabetes Care* 27 861–868. 10.2337/diacare.27.4.861 15047639

[B170] Tsochatzis E. Papatheodoridis G. V. Hadziyannis E. Georgiou A. Kafiri G. Tiniakos D. G. (2008). Serum adipokine levels in chronic liver diseases: association of resistin levels with fibrosis severity. *Scand. J. Gastroenterol.* 43 1128–1136. 10.1080/00365520802085387 18609175

[B171] Tworoger S. S. Mantzoros C. Hankinson S. E. (2007). Relationship of plasma adiponectin with sex hormone and insulin-like growth factor levels. *Obesity (Silver Spring)* 15 2217–2224. 10.1038/oby.2007.263 17890489

[B172] Ufkin M. L. Peterson S. Yang X. Driscoll H. Duarte C. Sathyanarayana P. (2014). miR-125a regulates cell cycle, proliferation, and apoptosis by targeting the ErbB pathway in acute myeloid leukemia. *Leuk. Res.* 38 402–410. 10.1016/j.leukres.2013.12.021 24484870 PMC4117580

[B173] Unsal M. Kara N. Karakus N. Tural S. Elbistan M. (2014). Effects of leptin and leptin receptor gene polymorphisms on lung cancer. *Tumor Biol.* 35 10231–10236. 10.1007/s13277-014-2293-2 25027400

[B174] van der Poorten D. Milner K. L. Hui J. Hodge A. Trenell M. I. Kench J. G. (2008). Visceral fat: a key mediator of steatohepatitis in metabolic liver disease. *Hepatology* 48 449–457. 10.1002/hep.22350 18627003

[B175] Vanni E. Bugianesi E. (2014). Obesity and liver cancer. *Clin. Liver. Dis.* 18 191–203. 10.1016/j.cld.2013.09.001 24274874

[B176] Villanueva A. Llovet J. M. (2014). Liver cancer in 2013: mutational landscape of HCC-the end of the beginning. *Nat. Rev. Clin. Oncol.* 11 73–74. 10.1038/nrclinonc.2013.243 24395088 PMC12261303

[B177] Vongsuvanh R. George J. Qiao L. van der Poorten D. (2012). Visceral adiposity in gastrointestinal and hepatic carcinogenesis. *Cancer Lett.* 330 1–10. 10.1016/j.canlet.2012.11.038 23201597

[B178] Wajchenberg B. L. (2000). Subcutaneous and visceral adipose tissue: their relation to the metabolic syndrome. *Endocr. Rev.* 21 697–738. 10.1210/edrv.21.6.0415 11133069

[B179] Wang J. Obici S. Morgan K. Barzilai N. Feng Z. Rossetti I. (2001). Overfeeding rapidly induces leptin and insulin resistance. *Diabetes Metab. Res. Rev.* 50 2786–2791. 10.2337/diabetes.50.12.2786 11723062

[B180] Wang T. Ma X. Peng D. Zhang R. Sun X. Chen M. (2016). Effects of obesity related genetic variations on visceral and subcutaneous fat distribution in a Chinese population. *Sci. Rep.* 6:20691 10.1038/srep20691 PMC4742921 26848030

[B181] Wang X. Lee S. O. Xia S. Jiang Q. Luo J. Li L. (2013). Endothelial cells enhance prostate cancer metastasis via IL-6→androgen receptor→TGF-β→MMP-9 signals. *Mol. Cancer. Ther.* 12 1026–1037. 10.1158/1535-7163.MCT-12-0895 23536722 PMC3782851

[B182] Watanabe N. Takai K. Imai K. Shimizu M. Naiki T. Nagaki M. (2011). Increased levels of serum leptin are a risk factor for the recurrence of stage I/II hepatocellular carcinoma after curative treatment. *J. Clin. Biochem. Nutr.* 49 153–158. 10.3164/jcbn.10-149 22128212 PMC3208009

[B183] Weisberg S. P. McCann D. Desai M. Rosenbaum M. Leibel R. L. Ferrante A. W. Jr. (2003). Obesity is associated with macrophage accumulation in adipose tissue. *J. Clin. Invest.* 112 1796–1808. 10.1172/JCI19246 14679176 PMC296995

[B184] Welzel T. M. Graubard B. I. Quraishi S. Zeuzem S. Davila J. A. El-Serag H. B. (2013). Population-attributable fractions of risk factors for hepatocellular carcinoma in the United States. *Am. J. Gastroenterol.* 108 1314–1321. 10.1038/ajg.2013.160 23752878 PMC4105976

[B185] Wieckowska A. Papouchado B. G. Li Z. Lopez R. Zein N. N. Feldstein A. E. (2008). Increased hepatic and circulating interleukins-6 levels in human non-alcohlic steatohepatitis. *Am. J. Gastroenterol.* 103 1372–1379. 10.1111/j.1572-0241.2007.01774.x 18510618

[B186] Winer D. A. Winer S. Shen L. Wadia P. P. Yantha J. Tsui H. (2011). B cell promote insulin resistance through modulation of T cells and production of pathogenic IgG antibodies. *Nat. Med.* 17 610–617. 10.1038/nm.2353 21499269 PMC3270885

[B187] Wisse B. E. (2004). The inflammatory syndrome: the role of adipose tissue cytokines in metabolic disorders linked to obesity. *J. Am. Soc. Nephrol.* 15 2792–2800. 10.1097/01.ASN.0000141966.69934.21 15504932

[B188] Wolin K. Y. Carson K. Colditz G. A. (2010). Obesity and cancer. *Oncologist* 15 556–565. 10.1634/theoncologist.2009-0285 20507889 PMC3227989

[B189] Wong Y. W. Cannell I. G. de Moor C. H. Hill K. Garside P. G. Hamilton T. L. (2008). The mechanism of micro-RNA-mediated translation repression is determined by the promoter of the target gene. *Proc. Natl. Acad. Sci. U.S.A.* 105 8866–8871. 10.1073/pnas.0800650105 18579786 PMC2449332

[B190] Wood I. S. de Heredia F. P. Wang B. Trayhurn P. (2009). Cellular hypoxia and adipose tissue dysfunction in obesity. *Proc. Nutr. Soc.* 68 370–377. 10.1017/S0029665109990206 19698203

[B191] Xie H. Li L. Zhu G. Dang Q. Ma Z. He D. (2015). Infiltrated pre-adipocytes increase prostate cancer metastasis via modulation of the miR-301a/androgen receptor (AR)/TGF-β/Smad/MMP9 signals. *Oncotarget* 6 12326–12339. 10.18632/oncotarget.3619 25940439 PMC4494941

[B192] Xie H. Lim B. Lodish H. F. (2009). MicroRNAs induced furing adipogenesis that accelerate fat cell developent are downregulated in obesity. *Diabetes Metab. Res. Rev.* 58 1050–1057. 10.2337/db08-1299 PMC2671055 19188425

[B193] Yamauchi T. Kamon J. Ito Y. Tsuchida A. Yokomizo T. Kita S. (2003). Cloning of adiponectin receptors that mediate antidiabetic metabolic effects. *Nature* 423 762–769. 10.1038/nature01705 12802337

[B194] Yang W. Lu Y. Xu Y. Xu L. Zheng W. Wu Y. (2012). Estrogen represses hepatocellular carcinoma (HCC) growth via inhibiting alternative activation of tumor-associated macrophages (TAMs). *J. Biol. Chem.* 287 40140–40149. 10.1074/jbc.M112.348763 22908233 PMC3504728

[B195] Yeh C. L. Cheng I. C. Hou Y. C. Wang W. Yeh S. L. (2014). Micro-RNA-125a-3p expression in abdominal adipose tissues is associated with insulin signaling gene expressions in morbid obesity; observations in Taiwanese, Asia. *Pac. J. Clin. Nutr.* 23 331–337. 10.6133/apjcn.2014.23.2.20 24901105

[B196] Yoshimoto S. Loo T. M. Atarashi K. Kanda H. Sato S. Oyadomari S. (2013). Obesity-induced gut microbial metabolite promotes liver cancer through senescence secretome. *Nature* 499 97–101. 10.1038/nature12347 23803760

[B197] Yu R. Kim C. S. Kwon B. S. Kawada T. (2006). Mesenteric adipose tissue-derived monocyte chemoattractant protein-1 plays a cruicial role in dipose tissue macrophage migration and activation in obese mice. *Obesity (Silver Spring)* 14 1353–1362. 10.1038/oby.2006.153 16988077

[B198] Zender L. Kubicka S. (2008). Androgen receptor and hepatocarcinogenesis: what do we learn from HCC mouse models? *Gastroenterology* 135 738–740. 10.1053/j.gastro.2008.07.034 18692055

[B199] Zhang Q. Xu D. Zhang M. Dong X. Dong H. Pan Q. (2016). Construction and analysis of an adipose tissue-specific and methylation-sensitive promoter of leptin gene. *Appl. Biochem. Biotechnol.* 10.1007/s12010-016-2162-0 [Epub ahead of print]. 27299919

[B200] Zhang W. Li C. Liu M. Chen X. Shuai K. Kong X. (2016). Aquaporin 9 is down-regulated in hepatocellular carcinoma and its over-expression suppresses hepatoma cell invasion through inhibiting epithelial-to-mesenchymal transition. *Cancer Lett.* 378 111–119. 10.1016/j.canlet.2016.05.021 27216981

[B201] Zhang Z. C. Liu Y. Xiao L. L. Li S. F. Jiang J. H. Zhao Y. (2015). Upregulation of miR-125b by estrogen protects against non-alcoholic fatty liver in female mice. *J. Hepatol.* 63 1466–1475. 10.1016/j.jhep.2015.07.037 26272872

[B202] Zhao J. Lawless M. W. (2013). Stop feeding cancer: pro-inflammatory role of visceral adiposity in liver cancer. *Cytokine* 64 626–637. 10.1016/j.cyto.2013.09.009 24120848

[B203] Zhou Y. Peng J. Jiang S. (2014). Role of histone acetyltransferases and histone deacetylases in adipocyte differentiation and adipogenesis. *Eur. J. Cell. Biol.* 93 107–107. 10.1016/j.ejcb.2014.03.001 24810880

